# Flocculation Mechanisms in *Brettanomyces bruxellensis*: Influence of ethanol and sulfur dioxide on *FLO* gene expression

**DOI:** 10.1016/j.crmicr.2025.100372

**Published:** 2025-03-07

**Authors:** Alessandra Di Canito, Roberto Foschino, Ileana Vigentini

**Affiliations:** Department of Biomedical, Surgical and Dental Sciences (DiSBIOC), Università degli Studi di Milano, Via della Commenda 10, 20122 Milan, Italy

**Keywords:** *Brettanomyces bruxellensis*, *Dekkera bruxellensis*, Flocculation, *Flo genes*, Response surface methodology, Yeast phenotype

## Abstract

The mechanisms underlying flocculation in *Brettanomyces bruxellensis*, unlike the well-characterized *FLO*-family gene regulation in *Saccharomyces cerevisiae*, remain largely unexplored. This study investigates the flocculant phenotypes of 99 *B. bruxellensis* strains, revealing that only a minority exhibits this clumping behavior and confirms its strain-dependent attitude. Focusing on two strains, CBS2499 (flocculant) and UMY321 (non-flocculant), genetic analysis uncovered polymorphisms and distinct allelic heterozygosity in the *FLO1* and *FLO11* genes, potentially linked to the phenotypic differences. To further examine these traits, Response Surface Methodology (RSM) was used to simulate oenological conditions, testing the impact of pH, ethanol, and sulfur dioxide (SO₂) levels on flocculation and gene expression. The findings revealed that environmental stressors, especially ethanol and SO₂, significantly increase the expression of *FLO1* and *FLO11* in CBS2499, indicating a regulatory role in flocculation under stress. These insights broaden our understanding of stress adaptation in *B. bruxellensis*, especially its survival strategies in wine environments. By elucidating factors influencing flocculation, this study contributes valuable knowledge for managing *B. bruxellensis* spoilage, potentially aiding in the development of targeted approaches to reduce its impact on wine quality.

## Highlights

• Flocculation in *Brettanomyces bruxellensis* is a strain-specific trait.
• *FLO1* and *FLO11* genes show variability, enhancing stress adaptation and persistence.
• Ethanol and SO₂ synergistically upregulate *FLO* genes in flocculant strains.
• Insights aid in managing *B. bruxellensis* spoilage via targeted winemaking strategies.

## Graphical abstract

**Figure:**
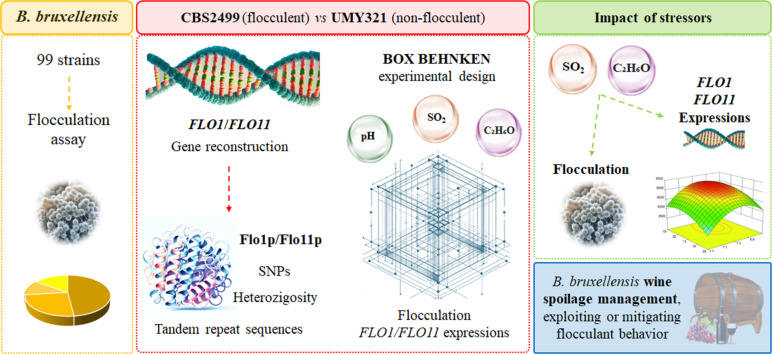

## 1 Introduction

All microorganisms, including yeasts, develop adaptation mechanisms to endure challenging environmental conditions. In *Saccharomyces cerevisiae*, in particular, a switch from unicellular to multicellular arrangement has been observed (Bouyx et al., 2021); one such multicellular growth strategy is flocculation, a phenomenon first described by Emil Hansen in the early 1900s (Vidgren and Londesborough, 2011). Flocculation is a reversible, asexual, calcium-dependent process of cell aggregation, mediated by lectin-like proteins, known as flocculins, which are present on the cell surface. These proteins facilitate the formation of flocs, which are large clusters of cells attached to each other that quickly sink to the bottom of the liquid medium while increasing the radius of the particle in suspension. Generally, the interaction between the flocculins and the protein receptor on the other cells is mediated by calcium ions (Vidgren and Londesborough, 2011; Tofalo et al. 2016). Flocculins, covalently linked to glucans in the cell wall through glycosylphosphatidylinositol (GPI) are encoded by the genes belonging to the *FLO* family ([Bibr bib0021][Bibr bib0020]), which show a high variability influencing the type and the intensity of flocculation (Vidgren and Londesborough, 2011). The recombination of internal repeats in these genes results in an increase in related protein size, which can alter the phenotype, such as the flocculant character. As evidenced in *S. cerevisiae* literature, *FLO1* and *FLO11* have been identified as the key regulators of flocculation and cell adhesion among all *FLO* family genes (Bayly et al. 2005). Specifically, the *FLO1* gene encodes for a lectin-like protein that is tightly associated with the cell walls and binds to the mannan carbohydrates of neighbouring cells; it plays a role in protecting cell aggregates from a variety of hostile conditions. The *FLO11* gene is one of the genes responsible for cell flocculation and the *flor* trait, regulating cell-cell adhesion, pseudohyphae formation and invasive growth (Bayly et al. 2005; Benitez et al., 2011; Stewart et al., 2018). Based on their flocculation mechanisms, *S. cerevisiae* strains are grouped in three phenotypic variants: *FLO1*, New*Flo* and *MI* (Mannose Insensitive) (Tofalo et al. 2016). In addition to the presence and structure of the *FLO* genes, the concentration of various molecules such as mannose, ethanol and sulphur dioxide in the growth medium are recognised as influencing the phenomenon. Other factors such as pH, available nitrogen, growth phase, may affect the phenotype, as a change in expressed proteins in the cell wall has been observed during the stationary phase (Smukalla et al. 2008; Tofalo et al. 2016).

In winemaking, the flocculant character of yeasts for primary fermentation is undesirable in order to avoid sluggish fermentation due to cell sedimentation, whereas it can be preferred for the refermentation step in sparkling wine production, as well as for industrial applications, where flocculation is useful to remove sedimented cells (Martìnez-Garcìa et al., 2020).

In this context, the study of the flocculant character can be extended to non-*Saccharomyces* yeasts to deepen our understanding of this phenotypic trait and explore its potential applications in addressing critical oenological issues or developing innovative technological approaches. While flocculation mechanisms have been extensively documented in *S. cerevisiae*, their characterization in non-*Saccharomyces* yeasts, such as *Brettanomyces bruxellensis*, remains limited. However, flocculant behavior in these microorganisms could represent a survival strategy under harsh environmental conditions, such as those encountered in winemaking processes. Understanding the genetic and physiological basis of flocculation in non-*Saccharomyces* yeasts would not only fill a significant knowledge gap but also provide valuable tools for microbiological control and the optimization of fermentation processes. *Brettanomyces bruxellensis* is a facultative anaerobic yeast, isolated from different sources including fruit peels, beer, wine, cheese, kombucha, kefir, tea, olives, sodas, and wooden barrels (Colomer et al. 2019). While contaminating and growing in wine, it generates unpleasant aromas associated with “horse sweat”, “leather”, “barnyard”, “medicinal”, “smoked” descriptors (Di Canito et al. 2021), well-known as “*Brett*” character. The cause of this disagreeable smell is due to the production of volatile phenols (4–vinylphenol, 4–vinylguiacol, 4–ethyl phenol, and 4–ethylguiacol) (Di Canito et al. 2021). The formation of ethyl phenols from vinyl phenols reduction is due to a distinctive enzymatic activity of *B. bruxellensis* species, which contains a Cu/Zn superoxide dismutase that functions as a vinyl phenol reductase. This is made possible by specific NAD(P)^+^/NAD(P)H binding sequences that are absent or altered in other wine-relevant yeast species (Granato et al., 2015). In addition, excessive acetic acid production, biofilm formation, product haze, gas production and surface adhesion are often attributed to the development of *B. bruxellensis* in spoiled wines (Di Canito et al. 2021). A peculiarity of this species is the great cell pleomorphism that is influenced by the growth phase and environmental conditions (Louw et al. 2016). Moreover, certain studies highlighted that *B. bruxellensis* cells can change morphology in response to stress conditions like the ones occurring in the winemaking (Serpaggi et al., 2012; Vigentini et al. 2013; Avramova et al. 2018). The flocculation, as well as the bioadhesion properties in *B. bruxellensis* strains (Le Montagner et al. 2023) appears to be a response to stress that seems to be strain-dependent in its nature, as observed in the case of *S. cerevisiae* (Soares, 2011).

Although it might be assumed that *B. bruxellensis* shares with *S. cerevisiae* the same mechanisms to flocculate, no published studies have yet directly examined this hypothesis. Understanding flocculation in *Brettanomyces bruxellensis* is essential as it plays a key role in its persistence and spoilage potential in winemaking, contributing to the production of undesirable volatile phenols. Additionally, flocculation may serve as a stress adaptation mechanism, allowing *B. bruxellensis* to survive harsh oenological conditions such as high ethanol and SO₂ concentrations. Moreover, while often undesirable in primary fermentation, flocculation can have technological relevance in secondary fermentation and industrial applications, where controlled cell aggregation facilitates yeast removal. Despite the lack of previous studies, investigating this phenomenon fills a critical knowledge gap and provides insights into the ecological and industrial significance of *B. bruxellensis* Therefore, the present study aims to investigate the flocculant character in an extended collection of *B. bruxellensis* strains. Furthermore, a genetic analysis was conducted to examine the polymorphisms in the *FLO* genes of selected strains and assess the impact of environmental conditions in a similar wine growth medium on their expression levels by using a Response Surface Methodology.

## 2 Materials and methods

### 2.1 Yeast strains, growth media and culture conditions

A collection of 99 *B. bruxellensis* strains, originating from diverse sources and geographical locations (Table S1), was investigated. Cells were stored in Yeast Peptone Destrose medium (YPD) (10 g/L yeast extract, 20 g/L peptone, 20 g/L glucose, pH 5.5) supplemented with 20 % (v/v) glycerol at −80 °C. The revitalization of the cells was carried out inoculating the glycerol stock at 1 % (v/v) in YPD broth and incubating at 25 °C in aerobic condition for 3 days.

### 2.2 Ca^2+^-dependent flocculation assay

The test was performed according to Rossouw et al. (2015). Briefly, colony formation was obtained in WL nutrient agar medium (Scharlab, Sentmenat, Barcelona, Spain) incubated at 25 °C in aerobic condition for 5 days. Few isolated colonies *per* each strain were inoculated in 5 mL of YPD medium and cultured at 25 °C until reaching the stationary phase. The OD_600__nm_ was determined by mixing 40 μL of the culture with 160 μL of 50 mM EDTA pH 8.0 and vortexing the suspension vigorously (Reading A). One mL of the culture was centrifuged at 20,000 g for 5 min, followed by a washing step in sterile demineralized water. The pellet was then resuspended in 1 mL of 40 mM CaCl_2_. Subsequently, the samples were vigorously vortexed and left for 60 s. A 40 μL sample was taken from just below the meniscus and mixed with 160 μL of a 40 mM CaCl_2_ solution. A second OD_600_ measurement was performed (Reading B).

The percentage of flocculation was then calculated using the following formula:

$$Flocculation\;\left(\%\right)=\frac{A-B}{A}\times 100$$

Three biological replicates were carried out. The results were submitted to one-way Analysis of Variance (Minitab® Statistical Software 22, Minitab Ltd., Coventry CV32TE United Kingdom) to infer the existence of significant difference among the strains. Subsequently, the post-hoc Tukey's test was conducted by setting a value of *p* < 0.05. Before proceeding with the ANOVA, the flocculation percentage data were converted to the corresponding arcsin of the square root values to match the assumption of a normal distribution of values.

### 2.3 Reconstruction of *FLO1* and *FLO11* gene sequences in CBS2499 and UMY321 strains

CBS2499 and UMY321 strains were selected for genetic analysis of flocculation-related genes due to their different flocculation abilities and the availability of their wholly sequenced genomes. The NCBI database was queried for sequence retrieval (Sayers et al. 2022) and the BLAST tool was employed to analyze the sequences of the selected strains, assessing their similarity as a percentage of homology with other deposited sequences (Camacho et al. 2009). *S. cerevisiae* S288C and *B. bruxellensis* AWRI1499 were used as reference strains. Genomic DNAs were extracted as described by Vigentini et al. (2012). For further analysis two genes of the *FLO*-family, *FLO1* and *FLO11*, were selected; the complete gene sequences in CBS2499 and UMY321 strains were determined by a primer walking PCR approach and the subsequent overlapping of generated fragments. To minimize the occurrence of sequencing errors, the forward primers were designed to be approximately 50–100 base pairs (bp) away from the reverse primer of the previous sequence. Additionally, the forward primer of the first upstream sequence and the reverse primer of the last downstream sequence were designed to be approximately 100 bp away from the beginning and end of the gene sequence, respectively, in order to safely obtain fragments containing the *Start* and *Stop* codons. All the designed primers are listed in Table 1. The PCR mixture included 1X Q5 DNA Polymerase buffer (New England, Biolabs, Ipswich, United States), 200 μM dNTPs (New England, Biolabs, Ipswich, United States), 0.5 μM of each primer, 0.02 U/µL Q5 Hot Start High-Fidelity DNA Polymerase (New England, Biolabs, Ipswich, United States) and approximately 100 ng of template DNA. The reaction was conducted in Mastercycler Nexus (Eppendorf, Hamburg, Germany), with the amplification proceeding as follows: initial denaturation at 98 °C for 5 min, followed by 35 cycles at 98 °C for 10 s, annealing temperature for 30 s, and extension at 72 °C for 1 min. A final extension step at 72 °C for 2 min was performed. The PCR products were purified with EuroClone® spinNAker purification kit (Euroclone, Milano, Italia) and sent to an external provider (Eurofins Genomics, Vimodrone, Italy) for sequencing. The complete reconstructed gene sequences are reported in Table S2.

**Table 1:** List of the primers used in this work.

Name	Sequence (5′ → 3′)	Tm ( °C)
**UMY321_FLO1_M0_Fw**	CTGTAATTACCCTGAAGTAG	53.7
**UMY321_FLO1_M0_Rv**	GTTCGTAAACTTTTACACCT	53.2
**UMY321_FLO1_M1_Fw**	AGGGTATGGCTGCTCCATATC	59.8
**UMY321_FLO1_M1_Rv**	CTGAAAGAATGCTTTGGATATCAGT	58.1
**UMY321_FLO1_M2_Fw**	AATACTTGGAGACGTTCTTG	53.2
**UMY321_FLO1_V_Fw**	TCAGCCTTCTGGCTCTGTTA	57.3
**UMY321_FLO1_V_Rv**	CCTCTTAGATATCAATAAAGTCTA	54.2
**UMY321_FLO11_M0_Fw**	AAGAGTTGTAATTACATGTTTAAG	52.5
**UMY321_FLO11_M0_Rv**	ATTTGGATTCTGCGACCAAT	53.2
**UMY321_FLO11_M1_Fw**	CTATTGGCCTAAATATATTTGAAAA	53.1
**UMY321_FLO11_M1_Rv**	GAACTAGAAGTAGAAGCAGTC	54.0
**UMY321_FLO11_M2_Fw**	ATCCACTTTGTCCACTTCAT	53.2
**UMY321_FLO11_M2_Rv**	GATGGTGTAGGAATCGAAATA	54.0
**UMY321_FLO11_M3_Fw**	TACAAAATCTAGTGTGAGTACC	54.7
**UMY321_FLO11_V_Fw**	TCAACAGTGCCATTCCATCT	55.3
**UMY321_FLO11_V_Rv**	AGAATAAAAAGTACGAAAATGAATC	53.1
**FLO1_CBS2499_qPCR_Fw**	AACTTATTGACGACTTGACTGAC	57.1
**FLO1_CBS2499_qPCR_Rv**	ACATCATCTCCTGAGGAGCC	59.4
**FLO11_CBS2499_qPCR_Fw**	TCACAAGCACAAAATTACCT	51.1
**FLO11_CBS2499_qPCR_Rv**	GGAGCAGTTGAACATGTTTC	55.2
**DbTUB_Fw**	GTATCTGCTACCAGAAACCAACC	60.7
**DbTUB_Rv**	CCCTCACTAACATACCAGTGGAC	62.4
**FLO1_alignment_Fw**	CGAATCAGGAAATGGATCGG	57.3
**FLO1_alignment_Rv**	TTTGAGTAGCTGCGGATGA	54.5
**FLO11_alignment_Fw2**	TAGTTCTACTTCATCAACAACATCAACTTCAT	61.8
**FLO11_alignment_Rv2**	AGCTCAAAGTGGAAGTACTACTCAAACTAG	64.0

### 2.4 Analysis of the *FLO1* and *FLO11* genes

Finch TV 1.4.0 program (Geospiza Inc - 2004) was used to analyse the pherograms of the amplified sequences and to validate the reconstructed allelic sequence of each *FLO* gene. The presence of single nucleotide polymorphisms (SNPs) in the two *B. bruxellensis FLO* genes was investigated at homo/heterozygous level. SNPs frequency was calculated as the ratio between the number of SNPs and the total number of analysed nucleotides. Specifically, the frequency of heterozygosity was calculated as the ratio between the number of heterozygous SNPs and the total number of SNPs. Expasy tool was the software employed for gene translation into proteins and the analysis of their amino acid composition (Duvaud et al. 2021). The ScanProsite section of Expasy was utilized to analyse the obtained proteins and search for functional domains within their sequences (De Castro et al. 2006). Tandem Repeat Finder tool (Benson, 1999) was used to identify tandem repeats within the obtained protein sequences, while JPRED4 (Drozdetskiy et al. 2015) was used to predict the secondary structures. Additionally, the obtained genes were compared with those deriving from the available complete genome sequenced of three strains of *B. bruxellensis* AWRI1499, UCD2041 and AWRI1613; alignments of the gene sequences were conducted using the Clustal Omega program (Madeira et al., 2024) to identify the most and least conserved regions. This analysis aimed to evaluate the variability of gene sequences and identify the most variable region to compare with those of nine other genetically different strains tested for their flocculation character (CBS73, CBS74, CBS1943, CBS2796, CBS4459, CBS5206, UMY309, UMY320 and UMY334). Primers were designed upstream and downstream the variable regions, targeting highly conserved ones, followed by amplification of the regions to be compared across strains. The PCR mixture and programs were performed as described in Section 2.3. Sequence alignments using Clustal Omega program were conducted to assess the percentage of similarities and gene polymorphisms among the various strains. Then, they were clustered using the maximum likelihood (ML) method implemented in MEGA version 11 (Tamura et al., 2021). The analysis employed the Jones-Taylor-Thornton (JTT) substitution matrix, included all sites, and incorporated a gamma distribution of mutation rates with a shape parameter optimized to 2. To assess the robustness of the inferred phylogeny, 100 bootstrap replicates were performed (Tamura et al. 2021).

### 2.5 Experimental design and response surface methodology

To simulate oenological conditions, yeast cells were cultivated in Simil-Wine Medium (SWM) containing: 2.5 g/L glucose, 2.5 g/L fructose, 5 g/L glycerol, 5 g/L tartaric acid, 0.5 g/L malic acid, 0.2 g/L citric acid, 1.4 g/ L-lactic acid, 1.7 g/L yeast nitrogen base w/o AA (Difco, Sparks, MD, United States), 1.5 g/L ammonium sulphate, 0.005 g/L oleic acid, 0.5 mL/L tween 80, 0.015 g/L ergosterol.

A Box-Behnken design (three variables at three levels) was used to assess flocculation and *FLO1/FLO11* expression across 15 experimental conditions (Table 4), including a triplicate control (Runs 13–15). SWM samples were prepared with varying pH (3.5, 4.0, 4.5), ethanol (5, 8.75, 12.5 % v/v), and molecular SO₂ (0, 0.125, 0.25 mg/L).

Sterilized SWM samples (by 0.2 μm filters) were inoculated with *B. bruxellensis* cells, prepared from YPD-grown cultures, washed, and resuspended in sterile demineralized water. Inoculation was standardized at OD_600__nm_ 0.1/mL in 25 mL, followed by incubation at 25 °C under static, airtight conditions. Cell growth was monitored daily, and once OD_600__nm_ reached 1, cells were collected (20 OD_600__nm_ equivalent), pelleted (20,000 × g, 5 min, 4 °C; Hettich, Rotina 380R, Tuttlingen, Germany), flash-frozen in liquid nitrogen, and stored at −80 °C for gene expression analysis.

Flocculation and gene expression data were analyzed using Minitab® Statistical Software 22. The data were analysed relative to the baseline, defined by the permissive condition (0 mg/L molecular SO₂, pH 4.5, and 5 % ethanol), which served as the reference for comparison. Response Surface Methodology (RSM) was applied to evaluate the effects of pH, ethanol, and SO₂. The model's fit was assessed by using the coefficient of linearity (R-squared), and ANOVA validated main, linear, and quadratic effects. Significant factors (*p* < 0.05) were identified using standardized t-statistics and visualized in response surface plots based on second-order polynomial equations.

### 2.6 RNA extraction and cDNA synthesis

*B. bruxellensis* CBS2499 cells were collected from the 15 experimental runs at a final concentration of 20 OD_600nm_. To prevent RNA degradation, biomass was quickly recovered by centrifugation at 20,000 g for 5 min at 4 °C and immediately frozen using liquid nitrogen. Total RNA extraction from the cell pellets was performed using the Presto Mini RNA Yeast Kit (Geneaid, New Taipei City, Taiwan) with slight modifications. In brief, cell lysis was achieved through mechanical disruption in 500 mL Buffer RB, 5 mL *β*-mercaptoethanol, and an isovolume of glass beads (425–600 mm, Sigma–Aldrich, Saint Louis, MO, United States). Three cycles of tissue disruption were carried out using the TissueLyser (Qiagen, Hilden, Germany) for 2 min at the maximum oscillation frequency, alternating with 1 min on ice. The supernatant was then centrifuged at 16,000 g for 3 min (Hettich, Tuttlingen, Germany). RNA concentrations were determined using the NanoDrop®ND-1000 Spectrophotometer (Wilmington, DE, USA), and the integrity of the RNA was verified through agarose gel electrophoresis under denaturing conditions using 1 % (v/v) formaldehyde. The RNA samples were stored at −80 °C until cDNA synthesis. Retrotranscription of RNA was performed using the QuantiTect Reverse Transcription Kit (Qiagen, Hilden, Germany). The cDNA samples were stored at −20 °C until they were used for the qPCR assays.

### 2.7 Gene expression analyses

The qPCR reactions were carried out in a Realplex^4^ Mastercycler EP Gradient Thermocycler (Eppendorf, Hamburg, Germany) using a reaction mix composed of SYBR Green Master-Mix (Thermo Fisher Scientific - Applied Biosystems), 200 nM-100 nM-50 nM forward and reverse primers (Eurofins genomics, Ebersberg, Germany), and a 10-fold dilution of cDNA. The used primers are listed in Table 1. The qPCR amplification cycle consisted of 40 repetitions of 95 °C for 30 s, 54 °C for 30 s, and 65 °C for 30 s. At the end of the reaction (95 °C for 15 s), a melting curve was generated by increasing the temperature from 60 to 95 °C with a step of 0.5 °C. All cDNA samples were run as technical duplicates in a 96-well plate (Eppendorf, Hamburg, Germany). For each gene, at least three decimal serial dilutions were prepared and stored at −20 °C in DNA tubes (Eppendorf, Hamburg, Germany). The amplification curves were analysed using Realplex software (Eppendorf, Hamburg, Germany). The 2^-ΔΔCt^ method, based on Livak and Schmittgen (2001), was applied to calculate the relative expression of *FLO1* and *FLO11*. The results were expressed as fold-changes, representing the increase or decrease in the expression value of the target gene, normalized against *DbTUB* expression (Valdetara et al. 2017) and compared to the calibrator expression (corresponding to the permissive growth condition with 0 mg/L mol. SO_2_, pH 4.5, and 5 % (v/v) ethanol).

## 3 Results

### 3.1 Assessment of the flocculant character

In this study, 99 genetically different strains of *B. bruxellensis*, sourced both from international and private (UMY) collections, were screened for their flocculation capacity using a calcium-dependent spectrophotometric assay. Previously, Rossouw et al. (2015), using the same testing protocol, reported that non-*Saccharomyces* strains exhibit a higher percentage of flocculation (ranging between 9 and 37 %) compared to two *Saccharomyces* spp. wine strains, which showed values below 7 %. Subsequently, although the experimental conditions and evaluation methods of flocculation differed, the latter observation was confirmed by Šuranská et al. (2016), who reported that, in a similarly sized collection of *S. cerevisiae* strains, 91 % exhibited weak flocculation.

Figure S1 illustrates the distribution of flocculant characteristics within the analysed collection, revealing a mean percentage of flocculation value of 14.7 % (actual strain values are shown in Table S1). Specifically, half of the strains (50 out of 99) exhibited a non-flocculant phenotype, characterized by values below 10 %, whereas over a quarter (27 out of 99) were classified as moderately flocculant, with flocculation values ranging from 10 % to 25 %. This latter finding highlighted that only 19 % of the population displayed the phenotypic trait of interest. Moreover, a significant difference in behaviour among strains was observed, which may be due to variations in the mechanisms of action of the *FLO* genes and the proteins they encode.

### 3.2 *FLO1* and *FLO11* gene analysis in CBS2499 and UMY321 strains

The CBS2499 and UMY321 strains, classified respectively as “flocculant” and “non-flocculant”, and of which the whole genomes have already been sequenced, were selected for a genetic analysis of flocculation-related genes. In particular, the *FLO1* and *FLO11* were considered as genes of interest due to their role in the expression of the flocculation trait, as highlighted by studies published by Tofalo et al. (2016) and Vidgren and Londesborough (2011) on various strains of *S. cerevisiae*.

First, genetic similarities in *FLO1* and *FLO11* genes were investigated using the genomes of *S. cerevisiae* S288C and *B. bruxellensis* AWRI1499 for comparison. The latter, as the first *B. bruxellensis* strain deposited in the database (Curtin et al., 2012), has already been used as a reference. Although both genes were identified in the genomes of CBS2499 and UMY321, the sequences available in the databases required further analysis to fill in the found gaps. Consequently, the reconstruction of the *FLO* gene sequences in the strains under study was carried out by a walking PCR approach, using the primers listed in Table 1. The obtained sequence fragments, as shown in Table S2, include underlined portions indicating the overlapping regions between adjacent sequences.

After assembly of the amplified fragments, the length of the *FLO1* gene was determined to be 2118 and 2358 bp for the CBS2499 and UMY321 strains, respectively, whereas the length of the *FLO11* gene was 2508 and 3024 bp for the CBS2499 and UMY321 strains, respectively. Then the sequences of the *FLO1* and *FLO11* genes found in the four considered strains were then aligned using the Clustal Omega program to evaluate the percentage of identity (Table 2) and gene polymorphisms.

**Table 2:** Percentages of identity resulting from the sequence alignments of *FLO1* and *FLO11* genes in four considered strains.

	S288C	AWRI1499	CBS2499	UMY321
	*FLO1* gene			
*S. cerevisiae* S288C	100.0	–	–	–
*B. bruxellensis* AWRI1499	44.0	100.0	–	–
*B. bruxellensis* CBS2499	46.1	87.8	100.0	–
*B. bruxellensis* UMY321	46.3	87.2	99.9	100.0
	*FLO11* gene			
*S. cerevisiae* S288C	100.0	–	–	–
*B. bruxellensis* AWRI1499	49.2	100.0	–	–
*B. bruxellensis* CBS2499	50.6	96.9	100.0	–
*B. bruxellensis* UMY321	50.9	97.1	96.1	100.0

The similarity resulting from the sequence alignments (Table 3) of the S288C *FLO1* gene were 44 % with AWRI1499 and 46 % with both UMY321 and CBS2499 strains. UMY321 and CBS2499 strains showed 87–88 % identity with AWRI1499 and almost 100 % each other.

**Table 3:** Allelic heterozygosis in *FLO1* (A) and *FLO11* (B) gene sequences of CBS2499 and UMY321 strains. SNP positions are calculated from the starting codon ATG. The heterozygosity positions shared between the strains are marked in bold character.

A)			
*FLO11* gene			
CBS2499 strain			
Position	Amino Acid Mutation	Mutation Type	Allelic heterozygosity (% at strain level)

**16 (T/C)**	Leu (L)/Leu (L)	Silent	0.68
**94 (G/A)**	Glu (E)/Lys (K)	Missense	
**99 (C/A)**	Ser (S)/Ser (S)	Silent	
**196 (G/A)**	Glu (E)/Lys (K)	Missense	
**468 (T/C)**	Ser (S)/Ser (S)	Silent	
**729 (G/A)**	Ser (S)/Ser (S)	Silent	
**777 (G/A)**	Ser (S)/Ser (S)	Silent	
**870 (G/A)**	Ser (S)/Ser (S)	Silent	
**963 (G/A)**	Ser (S)/Ser (S)	Silent	
**1059 (G/A)**	Ser (S)/Ser (S)	Silent	
**1509 (G/A)**	Thr (T)/Thr (T)	Silent	
**1622 (C/T)**	Ser (S)/Phe (F)	Missense	
**1666 (A/G)**	Ile (I)/Val (V)	Missense	
**1704 (G/A)**	Ser (S)/Ser (S)	Silent	
**1706 (A/G)**	Lys (K)/Arg (R)	Missense	
**1707 (G/A)**	Lys (K)/Lys (K)	Silent	
**2387 (C/T)**	Ser (S)/Leu (L)	Missense	

UMY321 strain			

**193 (G/T)**	Gly (G)/Cys (C)	Missense	0.83
**215 (C/G)**	Thr (T)/Ser (S)	Missense	
**232 (G/C)**	Asp (D)/His (H)	Missense	
**266 (T/G)**	Leu (L)/Trp (W)	Missense	
**271 (G/C)**	Val (V)/Leu (L)	Missense	
**277 (T/A)**	Ser (S)/Thr (T)	Missense	
**292 (A/T)**	Asn (N)/Tyr (Y)	Missense	
**293 (A/T)**	Asn (N)/Ile (I)	Missense	
**294 (T/C)**	Asn (N)/Asn (N)	Silent	
**303 (G/T)**	Glu (E)/Asp (D)	Missense	
**732 (G/A)**	Ser (S)/Ser (S)	Silent	
**2066 (G/C)**	Ser (S)/Thr (T)	Missense	
**2084 (G/T)**	Ser (S)/Ile (I)	Missense	
**2098 (T/A)**	Ser (S)/Thr (T)	Missense	
**2113 (G/T)**	Val (V)/Phe (F)	Missense	
**2138 (G/C)**	Ser (S)/Thr (T)	Missense	
**2176 (G/T)**	Gly (G)/Cys (C)	Missense	
**2198 (C/G)**	Thr (T)/Ser (S)	Missense	
**2201 (C/A)**	Ser (S)/Tyr (Y)	Missense	
**2226 (G/A)**	Ser (S)/Ser (S)	Silent	
**2232 (A/G)**	Ser (S)/Ser (S)	Silent	
**2237 (G/C)**	Arg (R)/Thr (T)	Missense	
**2259 (G/A)**	Thr (T)/Thr (T)	Silent	
**2283 (G/C)**	Ser (S)/Ser (S)	Silent	
**2291 (A/G)**	Gln (Q)/Arg (R)	Missense	

B)			
***FLO1* gene**			
**CBS2499 strain**			
**SNPs Position**	**Amino Acid Mutation**	**Mutation Type**	**Allelic heterozygosity (% at strain level)**

**3 (G/T)**	Met (M)/Ile (I)	Missense	1.46
**10 (T/C)**	Ser (S)/Pro (P)	Missense	
**23 (G/A)**	**Arg (R)/Lys (K)**	**Missense**	
**29 (T/G)**	Leu (L)/Trp (W)	Missense	
**40 (T/C)**	Ser (S)/Pro (P)	Missense	
**59 (T/G)**	Met (M)/Arg (R)	Missense	
**63 (T/G)**	Ala (A)/Ala (A)	Silent	
**66 (T/C)**	**Ala (A)/Ala (A)**	**Silent**	
**87 (T/G)**	**Gly (G)/Gly (G)**	**Silent**	
**100 (G/A)**	Glu (E)/Lys (K)	Missense	
**105 (T/G)**	Arg (R)/Arg (R)	Silent	
**868 (G/A)**	Glu (E)/Lys (K)	Missense	
**1322 (C/G)**	Thr (T)/Ser (S)	Missense	
**1340 (C/G)**	Ala (A)/Gly (G)	Missense	
**1343 (G/T)**	Ser (S)/Ile (I)	Missense	
**1386 (G/A)**	Ser (S)/Ser (S)	Silent	
**1387 (G/A)**	Gly (G)/Arg (R)	Missense	
**1388 (G/C)**	Gly (G)/Ala (A)	Missense	
**1978 (G/C)**	Val (V)/Ala (A)	Missense	
**1998 (G/T)**	Leu (L)/Phe (F)	Missense	
**2013 (T/C)**	Ala (A)/Ala (A)	Silent	
**2027 (C/T)**	Ser (S)/Phe (F)	Missense	
**2031 (C/A)**	Gly (G)/Gly (G)	Silent	
**2033 (C/G)**	Ser (S)/Cys (C)	Missense	
**2036 (T/A)**	Val (V)/Asp (D)	Missense	
**2037 (T/A)**	Val (V)/Val (V)	Silent	
**2044 (T/G)**	Tyr (T)/Asp (D)	Missense	
**2046 (C/G)**	Tyr (T)/Stop	Nonsense	
**2053 (G/A)**	Ala (A)/Thr (T)	Missense	
**2095 (T/G)**	Leu (L)/Trp (W)	Missense	

**UMY321 strain**			

**23 (G/A)**	**Arg (R)/Lys (K)**	**Missense**	0.30
**66 (T/C)**	**Ala (A)/Ala (A)**	**Silent**	
**87 (T/G)**	**Gly (G)/Gly (G)**	**Silent**	
**1497 (T/C)**	Ser (S)/Ser (S)	Silent	
**1503 (A/C)**	Ala (A)/Ala (A)	Silent	
**1515 (C/T)**	Val (V)Val (V)	Silent	
**1521 (C/A)**	Gly (G)/Gly (G)	Silent

**Table 4:** Box-Behnken experimental design conditions and relevant flocculation values (%) of CBS2499 and UMY321 strains.

Run	pH	Ethanol (% v/v)	molecular SO_2_ (mg/L)	CBS2499 strain Flocculation (%)	UMY321strain Flocculation (%)
1	3.5	8.75	0	52.4 ± 6.5	9.7 ± 2.3
2	3.5	8.75	0.250	53.5 ± 6.4	8.2 ± 1.5
3	4.5	8.75	0	73.1 ± 5.9	13.2 ± 1.4
4	4.5	8.75	0.250	35.4 ± 7.2	7.7 ± 2.8
5	4	5	0	69.8 ± 4.4	3.4 ± 0.4
6	4	5	0.250	28.9 ± 7.8	7.7 ± 2.7
7	4	12.5	0	35.7 ± 7.3	4.9 ± 1.2
8	4	12.5	0.250	80.2 ± 4.2	6.0 ± 1.3
9	3.5	5	0.125	63.6 ± 5.7	7.0 ± 1.9
10	4.5	5	0.125	49.3 ± 5.2	0.6 ± 0.2
11	3.5	12.5	0.125	84.9 ± 4.1	8.9 ± 3.8
12	4.5	12.5	0.125	30.7 ± 8.6	16.2 ± 2.1
13	4	8.75	0.125	72.6 ± 5.5	11.5 ± 1.4
14	4	8.75	0.125	71.1 ± 5.8	11.2 ± 1.1
15	4	8.75	0.125	74.3 ± 5.4	11.4 ± 3.6

Concerning the *FLO11* gene, S288C strain showed 49 % similarity with AWRI1499, 51 % with CBS2499 and UMY321. Meanwhile, the three *B. bruxellensis* strains exhibited approximately 96–97 % of sequence identity among them.

#### 3.2.1 Heterozygosis in *FLO1* and *FLO11* genes in CBS2499 and UMY321

The CBS2499 and UMY321 strains are diploid (Fournier et al. 2017). The reconstructed sequences of the *FLO1* and *FLO11* genes were subsequently examined to assess the presence of heterozygosity. In general, the sequence alignment of the strains CBS2499 and UMY321 showed a higher percentage of identity (99.86 %) in the *FLO1* gene than in the *FLO11* gene (96.08 %). However, the analysis revealed distinct differences between the two genes in allelic heterozygosity and mutation spectra. In the CBS2499 strain, the *FLO1* gene exhibited 30 SNPs, counting 8 silent and 21 missense mutations, which include a key position of a starting codon at 3 (Met→Ile), with an allelic heterozygosity of 1.46 %. Additionally, a nonsense mutation at position 2046 (Tyr→Stop) was identified, which could truncate the relate protein. In contrast, the UMY321 strain showed only 7 SNPs in the *FLO1* gene and an allelic heterozygosity of 0.30 %. Specifically, positions 66, 87, 1497, 1503, 1515 and 1521 were silent mutations, causing no change in the amino acids, while the polymorphism at position 23 was a missense substitution of an arginine for a lysine. As regards the *FLO11* gene, CBS2499 strain had 17 SNPs, resulting in a lower allelic heterozygosity of 0.68 %, and including 11 silent and 6 missense mutations. UMY321 exhibited 25 SNPs in the *FLO11* gene, with 6 silent and 19 missense mutations at positions and an allelic heterozygosity of 0.83 %. These findings indicate a higher mutation load and allelic heterozygosity in CBS2499 compared to UMY321, suggesting potential strain-specific differences in flocculation phenotypes. In Table 3 the results of the comparative analysis are reported.

#### 3.2.2 Flo1p and Flo11p domain identification and analysis of the protein sequences

**Fig. 1:**
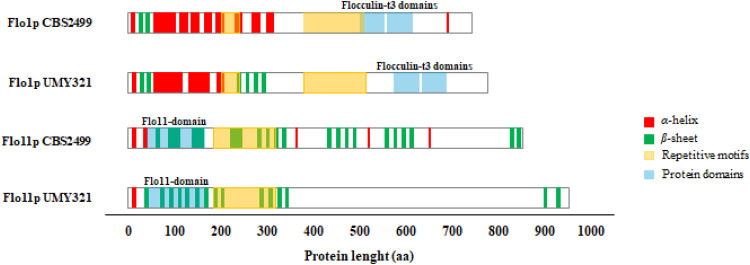
Summary of the characteristics of Flo1p and Flo11p in CBS2499 and UMY321 strains.

The translation of the reconstructed *FLO* genes into their respective proteins are summarized in Fig. 1. The analysis of the Flo1p sequence of both strains identified domains that were recognized as “Flocculin_t3 repeats”, located within the most conserved regions of the protein. These domains are characterized by a repetitive structure and are typically involved in mediating cell-cell interactions and adhesion (Goossens and Willaert, 2012). The examined sequences displayed features consistent with the Flo1p protein of *S. cerevisiae* (Goossens and Willaert, 2010), characterized by a high serine, threonine and glycine content. Particularly, Flo1p in CBS2499 strain had a length of 706 amino acids with 22.0 % serine, 11.8 % threonine and 9.1 % glycine, whereas in UMY321 it had a length of 762 amino acids with 24.0 % serine, 12.2 % threonine and 9.8 % glycine. Repeats of 4 amino acids are numerous and frequently distributed throughout the sequences. Some examples include the SSSS, SSSV and GSGS motifs that may indicate some structural flexibility or a role in regulatory interactions such as phosphorylation or other post-translational modifications. With regard to the Flo11p sequences of the strains the analysis revealed a domain recognized as the “*FLO11*-superfamily”, which is located in the initial and most conserved regions. From the N-terminal side, a 176 amino acid domain with β-sheet regions was identified in both sequences of the two strains, corresponding to a fibronectin Type III-like adhesin found in various ascomycetes (Kraushaar et al., 2015). In *S. cerevisiae* this Flo11pA domain, consisting of around 187 amino acids, is O-glycosylated and this structural complexity is essential for its homotypic interactions, supporting the adhesive role of Flo11p in yeast biofilm formation (Goossens and Willaert, 2012). Specifically, the Flo11p protein from CBS2499 was 835 amino acids long with 30.0 % serine and 22.9 % threonine, while that from UMY321 showed a length of 946 amino acids with 31.9 % serine and 23.7 % threonine. In the CBS24999 strain, 80 repeats of different lengths were present, whereas in the UMY321 strain 58 repeats were identified, although the sequence was longer of 111 amino acids. Many short repetitive motifs, such as STSST, STTSS and TSSSS suggested that the protein may have a complex and dynamic structure with multiple sites for phosphorylation and glycosylation. The Flo1p and Flo11p proteins, associated with the CBS2499 and UMY321 yeast strains, exhibit distinct secondary structural properties. Flo1p is predominantly composed of α-helices, suggesting its involvement in strong cell-cell adhesion, in contrast, Flo11p shows a β-sheet-rich structure suggesting a key role in adhesion to surfaces and biofilm formation.

### 3.3 Potential correlation between the variability of *FLO1* and *FLO11* gene sequences and flocculant character

In order to investigate whether the observed sequence variability might influence the flocculant character, the most variable regions of *FLO1* and *FLO11* were identified by sequence alignments of the reconstructed genes in CBS2499 and UMY321 strains with those of the genes of strains AWRI1499, AWRI1613 and UCD2041 already available in the NCBI database. The variable regions selected were approximately 352–372 bp for *FLO1* and 533–1370 bp for *FLO11*, indicating that while *FLO1* maintains sequence stability, *FLO11* may offer a greater range of sequence flexibility. The selected regions were also examined through the amplification of the DNA of the CBS73, CBS74, CBS1943, CBS2796, CBS4459, CBS5206, UMY309, UMY320 and UMY334 strains and subsequently sequenced. The obtained sequences were then aligned with those of the aforementioned strains. The percentages of identity of the variable regions within *FLO* genes found in different *B. bruxellensis* strains are reported in Table S3.

Concerning the *FLO1* gene, AWRI1499 exhibited the lowest degree of similarity, with a range of 77.1 % to 79.6 %, indicating a relative divergence from other strains. In contrast, CBS2499, UMY321, AWRI1613, CBS2796, CBS74, CBS4459, UMY334 and UMY320 revealed near-uniform scores of 100 %, with the exception of CBS5206 and UMY309, which showed slightly lower values of 94.9 % and 94.5 %, respectively. The UCD2041 strain demonstrated a high level of alignment with CBS73 and CBS1943 with an identity value of 98.9 %, suggesting a close genetic relationship. Finally, CBS5206 and UMY309 shared a 97.5 % similarity, while lower values were found when comparing the sequences with those of the other strains, ranging from 78.2 % to 95.4 %.

For the *FLO11* gene, the CBS73 strain exhibited the highest degree of similarity with CBS74 (99.8 %), CBS2499 (99.5 %), CBS2796 (99.5 %), UMY309 (99.5 %) and CBS1943 (99.1 %). Other noteworthy pairings included CBS2796 strain that showed elevated scores of identities with UMY309 (99.6 %) and CBS1943 (99.4 %). CBS2499 and CBS1943 strains also demonstrated a strong alignment (99.4 %). In contrast, CBS4459 revealed the lowest level of similarity with UMY320 (68.4 %), UMY321 (69.5 %) and UMY334 (72 %) indicating a notable divergence from those. AWRI1499 also exhibited moderate similarity with various strains, including CBS4459 (70.6 %) and UMY320 (75.8 %). These results confirmed that the *FLO1* gene has a higher conserved sequence compared to the *FLO11* gene, which displayed a higher level of variability.

**Fig. 2:**
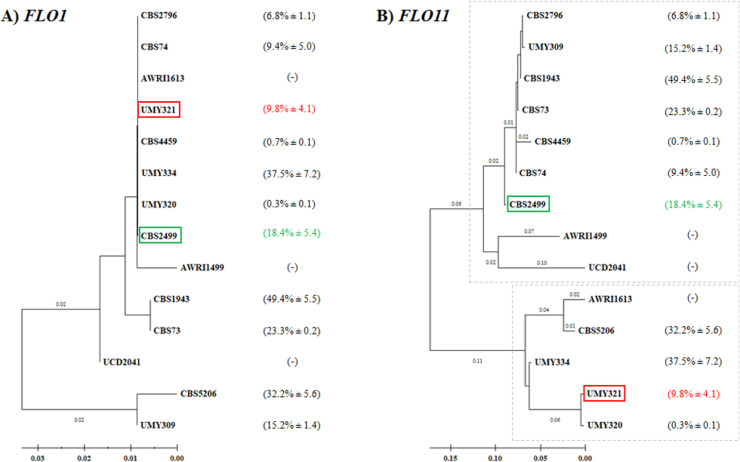
Phylogenetic analysis of the selected strains based on the alignments of the most variable regions in *FLO1* gene (A) and *FLO11* gene (B) sequences; the percentage of flocculence related to each strain are reported in brackets. The branch lengths are shown in the figures if they exceed 0.009 for the *FLO1* gene and 0.045 for the *FLO11* gene. The two clusters identified for the *FLO11* gene are highlighted with a square.

A cluster analysis was carried out on the most variable regions for each of the two examined genes (Fig. 2). The dendrogram generated by the analysis of *FLO1* gene revealed a large cluster comprising twelve strains, eight of which exhibited a very high level of similarity; conversely, a small group was formed by the CBS5206 and UMY309 strains, which displayed a notable degree of differentiation (Fig. 2A). The dendrogram produced by the elaboration of *FLO*11 gene sequences showed a clear grouping in two clusters (Fig. 2B): the first one was comprised of CBS2796, UMY309, CBS1943, CBS73, CBS4459, CBS74, CBS2499, AWRI1499 and UCD2041, while the second consisted of AWRI1613, CBS5206, UMY334, UMY321, and UMY320, without any resulting relation with the clustering found with *FLO1* gene. Furthermore, the phylogenetic distribution did not appear to correlate with either the source of isolation, geographic origin, or the flocculence capability.

### 3.4 Effect of oenological conditions on flocculation capability of CBS2499 and UMY321 strains

To evaluate the influence of the wine environment on the flocculant character and the expression of two *FLO* genes, a Box-Behnken experimental design with three factors (pH, ethanol content and molecular SO_2_ level) was performed. For both strains, the most permissive SWM conditions permitted the attainment of the predetermined OD within three days, whereas runs characterized by more restrictive factor values exhibited growth delays up to 13 days.

However, the results showed different outcomes for the two strains (Table 5).

**Table 5:** Regression equations which fitted to the data of the Box-Behnken experimental design. Factors are pH (A), ethanol % (v/v) (B) and molecular SO_2_ (mg/L) (C). The second-order equations show main (A, B and C), linear (AB, AC and BC) and quadratic effects (AA, BB and CC). Coefficients are the regression coefficients for the considered variable. R-Squared statistic indicates that the model as fitted explains a certain % of the variability in the considered variable.

Variable (*y*)	Regression model equation	R^2^ (%)
Flocculation of CBS2499 strain	*y* = −6.27 + 3.09 *A* + 0.278 *B* + 3.79 C - 0.318 AA - 0.00541 BB - 7.55 CC - 0.0586 AB - 1.60 AC + 0.475 BCE	82.2
Flocculation of UMY321 strain	*y* = −0.26 + 0.28 A - 0.020 *B* + 2.17 C - 0.075 AA - 0.00708 BB - 3.21 CC + 0.0402 AB - 0.257 AC - 0.0381 BCE	70.8
*FLO1* gene expression in CBS2499 strain	*y* = −0,25 + 0,43 *A* + 0,108 B - 12,66 C - 0,043 AA + 0,00,666 BB + 15,33 CC - 0,0533 AB + 1656 AC + 0,288 BCE	98.1
*FLO11* gene expression in CBS2499 strain	*y* = −21.8 + 9.1 *A* + 0.440 *B* + 29.9 C - 0.71 AA + 0.0686 BB - 78.2 CC - 0.325 AB - 2.41 AC - 0.363 BCE	90.0

**Fig. 3:**
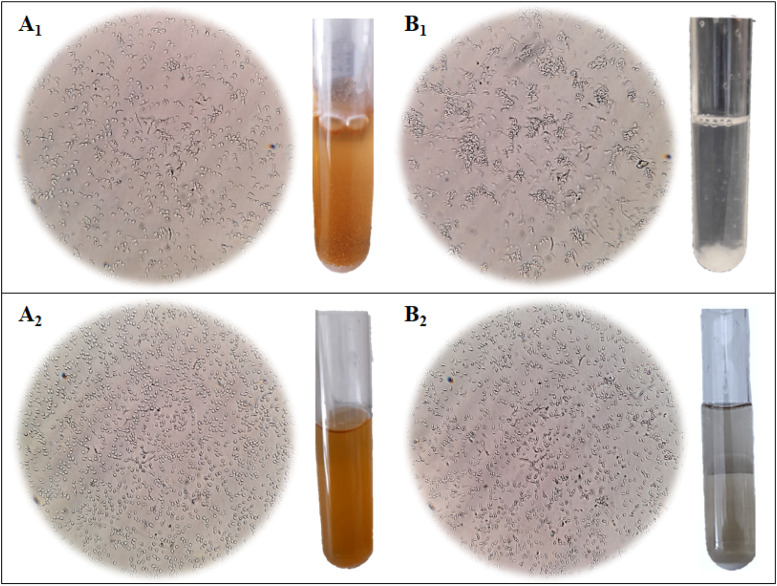
Microscopical observation of yeast cells (400 X) and tube images of related cultures. A_1_) CBS2499 strain grown in YPD; A_2_) UMY321 strain grown in YPD; B_1_) CBS2499 strain grown in SWM with pH = 4.0, ethanol 12.5 % (v/v) and 0.125 mg/L molecular SO_2_; B_2_) UMY321 strain grown in SWM with pH = 4.0, ethanol 12.5 % (v/v) and 0.125 mg/L molecular SO_2_.

For CBS2499, growth in the wine-like medium led to a significant increase in flocculation compared to the nutrient-rich broth. Specifically, the flocculation values increased from 18.4 % in YPD to 58.4 % in SWM, as an average, with a flocculation percentage in SWM between 28.9 % and 84.9 % across the different runs. On the contrary, UMY321 strain did not exhibit any change in behaviour, demonstrating to be a non-flocculant strain despite the variation of the tested oenological factors. Indeed, the flocculation percentage was 9.8 % in YPD and remained at an average of 8.3 % in SWM. In Fig. 3, the phenotypical changes of the two strains in YPD and SWM media can be observed.

Data from the 15 runs were processed using Minitab® statistical software 22. The regression equations, generated through the statistical processing of the data based on the proposed model, and their respective fit values expressed as R-Squared (%), are presented in Table 5.

**Fig. 4:**
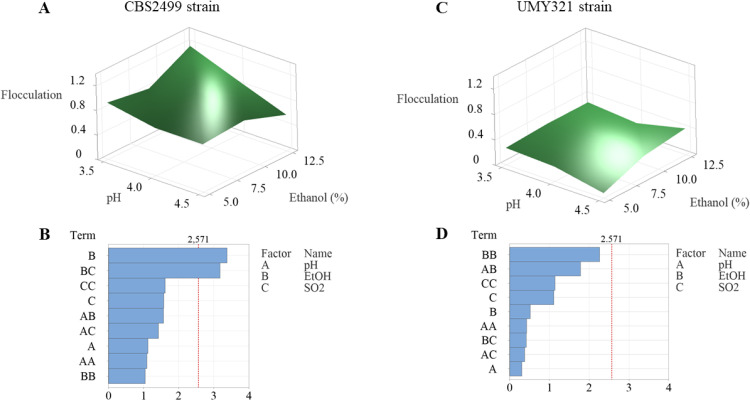
Response Surfaces fitted to experimental points of flocculation and relevant standardized Pareto charts for each variable. The line drawn on the diagram shows when an effect is statistically significant (α = 0.05). (A) Response surface of flocculation for CBS2499 strain as function of pH and ethanol % (v/v); (B) Pareto chart of standardized effects for CBS2499 strain; (C) response surface of flocculation for UMY321 strain as function of pH and ethanol % (v/v); (D) Pareto chart of standardized effects for UMY321 strain. Data used to generate the response surfaces refer to a molecular SO_2_ concentration of 0.125 mg/L.

The response surfaces constructed from the experimental points obtained from the flocculation tests in SWM for the CBS2499 strain (panel A) and the UMY321 strain (panel C) are shown in Fig. 4A and 4C, respectively. These surfaces illustrate the effects of variations in pH and ethanol concentration. Panels B and D display the corresponding Pareto charts. For CBS2499 strain, ethanol proved to be the main factor determining a significant change in phenotype (*p*-value = 0.020), as well as its interaction with molecular SO_2_ (*p*-value = 0.025). Conversely, the ANOVA of the data showed no significant effect for UMY321 strain: flocculation, which is moderately pronounced for this yeast, was not influenced by the environmental factors under investigation. Consequently, UMY321 strain was not included in the evaluation of the impact that pH, ethanol and molecular SO_2_ level could exert on *FLO* genes expression.

### 3.5 Effect of oenological conditions on *FLO1* and *FLO11* genes expression in CBS2499

The RSM approach, using the outcomes of the Box-Behnken experimental design, allowed to investigate the influence of pH, molecular SO₂ and ethanol variables on the expression of the two considered *FLO* genes. Real-time qPCR assays were conducted on cDNA samples derived from RNA extracted from CBS2499 cells grown in wine simulated environment. All the assays yielded amplification curves within the optimal sensitivity range of the qPCR (20–30 C_T_ values) and showed good reproducibility through all the tests. The calibrator gene *DbTUB* demonstrated consistent gene expression (C_T_ value of 23.4 ± 1.7) across the 15 conditions evaluated, confirming its reliable role as a housekeeping gene for *B. bruxellensis*, as previously documented by Valdetara et al. (2017).

**Fig. 5:**
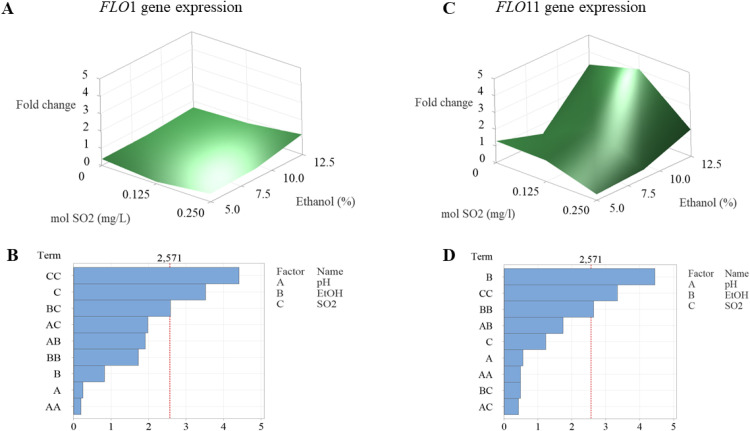
Response surfaces fitted to experimental points of *FLO* genes expression in CBS2499 strain and relevant standardized Pareto charts for each variable. The line drawn on the diagram shows when an effect is statistically significant (α = 0.05). (A) Response surface of *FLO*1 gene expression as function of molecular SO_2_ (mg/L) and ethanol % (v/v); (B) Pareto chart of standardized effects for *FLO*1 gene expression; (C) response surface of *FLO11* gene expression as function of molecular SO_2_ (mg/L) and ethanol % (v/v); (D) Pareto chart of standardized effects for *FLO11* gene expression. Data used to generate the response surfaces refer to a pH value of 4.0.

The calculated fold change values (2^-ΔΔCt^) for the gene of interest are reported in Table S4, while the response surfaces constructed from the experimental data on *FLO* gene expression in SWM are shown in Fig. 5.

The *FLO1* gene was upregulated compared to a permissive growth condition, with its highest expression (1-fold change) observed under a combination of 0.25 mg/L molecular SO_2_, pH 3.5/4, and 12.5 % (v/v) ethanol. The expression of *FLO1* (Fig. 5A and 5B) was significantly influenced by SO_2_ concentration, both at the linear (*p* = 0.017) and quadratic (*p* = 0.007) levels, as well as by its interaction with ethanol (*p* = 0.049). The high R-squared value (98.1 %) indicated a strong fit of the model to the experimental data (Table 6).

Regarding *FLO11* (Fig. 5C and 5D), its expression maximized (4-fold change) at 0.125 mg/L molecular SO_2_, pH 4.0, and 12.5 % (v/v) ethanol. Among the tested factors, ethanol was identified as the main driver of its increased expression, exhibiting both a main effect (*p* = 0.007) and a quadratic effect (*p* = 0.046). Additionally, a significant quadratic effect was observed for molecular SO_2_ (*p* = 0.020). The model demonstrated a good fit with the experimental results obtained in the wine-like medium trials (R² = 90.0 %, Table 6). Finally, the pH factor was found to have no impact on the expression of the two *FLO* genes under investigation.

## 4 Discussion

In *S. cerevisiae*, the *FLO*-family genes encode cell wall proteins that are essential for the flocculant phenotype. Flo1p is a protein that protrudes on the external cell surface to generate Ca^++^-dependent cell-cell adhesive interactions (Matheson et al. 2017) by forming reversible aggregates (flocs) through the binding of mannose residues in the cell walls of neighbouring cells. On the other hand, Flo11p, which contains a fibronectin Type III-like domain, is associated with adherence to solid surfaces, increased hydrophobicity, invasive growth, pseudohyphae development, and foam formation (Bayly et al. 2005; De Figuereido et al. 2021; Kraushaar et al., 2015). It is also involved in homotypic interactions, supporting the adhesive role of Flo11p in mat and air–liquid biofilm (*flor*) formation (Goossens and Willaert, 2012).

Despite *B. bruxellensis*, the most widespread spoilage yeast in wine, is known for its ability to adhere to working surfaces and oak barrels, the understanding of the role played by the *FLO* genes remains limited. Actually, as confirmed by our results, the flocculant phenotype appears to be a strain-specific calcium-dependent phenomenon in this species.

After a detailed reconstruction of two *FLO* genes in the CBS2499 and UMY321 strains, we investigated their allelic heterozygosity, which is recognised to be advantageous in increasing the evolutionary potential of a yeast population. Hellborg and Piškur (2009) first examined the heterozygosity in the species *B. bruxellensis* and found that in the diploid CBS2499 strain the distribution of polymorphic sites within the coding and non-coding regions was 0.4 % and 1.0 %, respectively. According to Curtin and colleagues (2012), the level of heterozygosity observed in the genome of the triploid *B. bruxellensis* strain AWRI1499 was higher, with a median of 27 single nucleotide polymorphisms per 1000 nucleotides. However, this SNP density was not uniform, with an average of 1.9 % of SNPs within predicted ORFs compared to those observed for the whole genome, of which only 0.7 % produced non-synonymous amino acid substitutions. Later, Fournier et al. (2017) sequenced the genome of the diploid UMY321 strain confirming a calculated averaged heterozygosity of 0.6 %, whereas some specific regions showing loss of heterozygosity. Our analysis yielded a similar outcome, but significant differences in allelic heterozygosity were found in the examined genes for the two strains. Regarding the *FLO1* gene, the CBS2499 strain showed a higher heterozygosity with 1 % missense SNPs compared to the UMY321 strain which had <0.1 % missense SNPs and an additional region of 240 bp. Conversely, the examination of the *FLO11* gene gave a different result, as the CBS2499 strain disclosed a lower heterozygosity (0.2 % missense SNPs) compared to the UMY321 strain, which showed 0.6 % missense SNPs and was 516 bp longer. A difference in heterozygosity between strains for the *FLO1* and *FLO11* genes can offer an adaptive advantage by enhancing phenotypic plasticity.

The analysis of the *FLO1* gene sequences detected several repeats in both strains. Moreover, the associated amino acid sequences showed features consistent with the Flo1p protein, characterised by a high content of serine allowing potential phosphorylation or O-linked glycosylation. The sequences comparison and the cluster analysis that we performed on the most variable regions of the *FLO1* gene disclose a marked stability in eleven strains of *B. bruxellensis* showing sequence identity greater than 99 %. This is consistent with previous findings in *S. cerevisiae* that emphasize the importance of conserved domains in ensuring the functional stability of the gene (Linder and Gustafsson, 2008; Kraushaar et al., 2015). Among the remaining strains, one exhibits a notable divergence from the others, as evidenced by a very dissimilar sequence, whereas the other two strains separate from the main group, yet they share a sequence similarity of 97.5 %.

In the *FLO11* gene, as pointed out by Fidalgo et al. (2008) in *S. cerevisiae*, the repeat is unstable and presents length variations that differentially affect Flo11p functions, generating combinatorial diversity that supports rapid adaptation. Zara et al. (2009) confirmed that the *FLO11* sequence is highly polymorphic in different strains of *S. cerevisiae* and that there was a significant correlation between the length of *FLO11* gene and the ability to form biofilms. Although the Flo11p protein of the CBS2499 strain was considerably shorter, it exhibited approximately one-third more repeats with different lengths than those observed in the UMY321 strain. The analysis of the most variable regions of the *FLO11* gene in the fourteen *B. bruxellensis* strains considered in this work revealed a significant reduction in sequence conservation respect *FLO1* gene, showing higher variability across both flocculant and non-flocculant strain. As previously observed in *S. cerevisiae* (Goossens and Willaert, 2012; de Figueiredo et al., 2021), the pronounced gene-length polymorphisms and domains with diverse repetitive structure suggest that this protein is strain-specific. Moreover, the presence of serine and threonine-rich regions is also indicative of potential regulation by post-translational modifications, thereby underscoring its potential role in influencing the responses to environmental stressors. In addition, the ethanol concentration in the growth medium may alter the hydrophobicity of the cell wall and could exert a slight chaotropic effect on the hydrophilic regions of the proteins that jut into the liquid phase.

In the absence of an evident correlation between the genetic sequences and flocculation phenotype, it can be postulated that environmental conditions can exert a pivotal influence on the expression of *FLO* genes in *B. bruxellensis.* This hypothesis is supported by previous research on *S. cerevisiae* (Vidgren and Londesborough, 2011; Soares, 2011). In particular, fluctuations in stress factors such as pH, ethanol and sulphur dioxide may act to modulate the transcriptional or post-transcriptional regulation of flocculence-associated genes.

Thus, with the aim to assess the impact of oenological factors and their interactions on flocculation and the expression of the *FLO1* and *FLO11* genes in *B. bruxellensis*, a RSM approach was used. The first finding was that the UMY321 strain, identified as non-flocculant through the preliminary screening, has subsequently confirmed its character without showing any alteration in the phenotype despite the different medium composition in the runs of DoE. On the contrary, the CBS2499 strain demonstrated a significant variation in flocculation rate as response to changes in ethanol content and the relative interaction with molecular SO_2_. This observation prompted further investigation into this strain by looking at the expression patterns of the two specific genes to explain the influence of the considered factors. As second outcome, the results obtained with the Box-Behnken experimental design proved that the expression of the *FLO1* gene in CBS2499 strain is significantly induced by the presence of SO_2_ with a positive interaction with the alcohol content, although the fold change was relatively low. Even more, the expression of the *FLO11* gene is strongly affected by ethanol and molecular SO₂ levels in the growth medium, with a markedly high fold change as the former factor increases. It can be concluded that these stress factors exert an up-regulating effect on the investigated genes, as already found in *S. cerevisiae* (Vidgren and Londesborough, 2011;, Soares, 2011). In particular, *FLO1* is recognised as the key gene controlling flocculation and its expression increases with the concentration of ethanol in the environment. Indeed, Smukalla and collaborators (2008) demonstrated that ethanol acts as a *quorum-sensing* molecule in the *FLO1*-mediated flocculation, inducing strong flocculation in the *S. cerevisiae* EM93 strain, thereby enhancing stress resistance and significantly improving cell survival rates. Additionally, Soares and colleagues (2011) affirmed that ethanol enhances yeast flocculation by reducing electrostatic repulsion and promoting binding interactions, though its impact on *FLO* gene expression varies depending on ethanol concentration and yeast strain.

A review of the literature reveals no prior studies that have addressed the expression of *FLO* genes in *B. bruxellensis*. Le Montagner et al. (2023, 2024) have recently investigated the adhesion phenomenon in *B. bruxellensis* by studying intraspecific variations in the physicochemical properties of the cell surface, as well as the influence of abiotic and biotic factors. In our experimental conditions, the analysis of *FLO1* and *FLO11* gene expression unveil as the flocculation is associated with response to stress caused by ethanol and sulphur dioxide, while changing the pH seems to have no effect, at least in the range of values tested. *B. bruxellensis* strains with flocculant behaviour would use this trait to form a multicellular structure to increase the chances of persistence in adverse conditions by increasing the expression of the *FLO* genes. Considering the main findings on the role of SO₂, although it is a narrow strategy to control volatile phenols production (Valdetara et al., 2017), higher concentrations can potentially limit the expression of the *FLO11* gene, thereby reducing cell adhesion and biofilm formation. This effect could help to manage *Brettanomyces* persistence across wine vintages.

## 5 Conclusions

Based on the findings, this study highlights the complex interplay between genetic and environmental factors influencing the flocculant phenotype in *Brettanomyces bruxellensis*. A higher heterozygosity in *FLO* genes may allow for a broader range of responses to environmental stimuli, such as varying adhesion, flocculation, or biofilm formation, thereby increasing the strain's ability to thrive under diverse or fluctuating conditions. Although no direct correlation between genetic sequences and flocculation was observed, the FLO1 and FLO11 genes structures affected the strain's ability to adapt to stressors such as ethanol and SO2, since they have been observed to modify gene expression, thus improving traits related to flocculation and cell adhesion.

The outcome of this work is that in aged wine and barrels, *B. bruxellensis* strains with flocculant character and increased adhesiveness are able to better survive, spread and persist in the cellar environment over time due to the selective pressure of ethanol and sulphur dioxide. Future research should focus on validating these findings through functional analyses of *FLO1* and *FLO11* mutants under winemaking conditions, assessing the direct impact of heterozygosity differences on flocculation. Additionally, transcriptomic and proteomic studies could provide deeper insights into regulatory networks influencing cell adhesion and stress response. From a technological perspective, this knowledge could support the development of targeted approaches to control *B. bruxellensis* spoilage, such as precision fermentation strategies that exploit or mitigate flocculant behavior. Moreover, understanding the genetic basis of adhesion and biofilm formation may inform strategies for biocontrol or novel yeast applications in industrial fermentation processes. These insights contribute valuable knowledge for managing *B. bruxellensis* spoilage in winemaking.

## Declaration of competing interest

The authors declare that they have no known competing financial interests or personal relationships that could have appeared to influence the work reported in this paper.

## Data Availability

Data will be made available on request.

## Appendix Supplementary materials

Image, application 1
