# Supplementary material for: Flocculation Mechanisms in Brettanomyces bruxellensis: Influence of ethanol and sulfur dioxide on FLO gene expression
Source: Curr Res Microb Sci. 2025 Mar 7;8:100372. doi: 10.1016/j.crmicr.2025.100372 (PMC11979396; doi:10.1016/j.crmicr.2025.100372)
Supplement: Supplementary file 1 [file mmc1.docx]

**Supplementary materials**

**Figure S1.** Box plot represents the distribution of the flocculant character in 99 *B. bruxellensis* strains


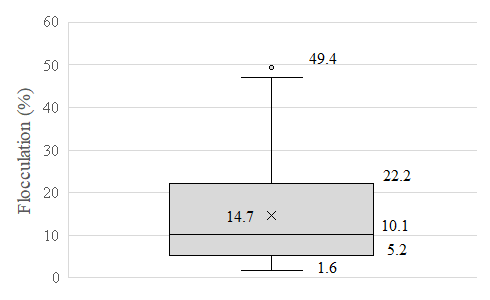


**Table S1.** List of *Brettanomyces/Dekkera* *bruxellensis* strains analysed in this work, with the corresponding source of isolation, geographical origin and the flocculation character expressed in percentage (mean and standard deviation).

| **Strain** | **Source of isolation** | **Geographical origin** | **Flocculation (%)** |
| --- | --- | --- | --- |
| CBS73 | Grape must | France | 23.3±0.2 |
| CBS74 ^T^ | Lambic beer | Belgium | 9.4±5.0 |
| CBS1940 | Sour wine | France | 22.2±2.5 |
| CBS1941 | Sour wine | France | 6.1±1.5 |
| CBS1942 | Sour wine | France | 11.4±3.8 |
| CBS1943 | Sour wine | France | 49.4±5.5 |
| CBS2336 | Wine | France | 18.8±2.2 |
| CBS2499 | Wine | France | 18.4±5.4 |
| CBS2547 | Sour wine | France | 33.9±3.5 |
| CBS2796 | Sparkling Mosselle wine | Germany | 6.8±1.1 |
| CBS2797 | Bordeaux wine | France | 4.3±0.9 |
| CBS4459 | Dry white wine | South Africa | 0.7±0.1 |
| CBS4480 | Dry white wine | South Africa | 4.7±2.6 |
| CBS4481 | Champagne | South Africa | 9.0±1.1 |
| CBS4482 | Sherry | South Africa | 11.4±4.9 |
| CBS4601 | Wine | South Africa | 4.6±1.8 |
| CBS4602 | Wine | South Africa | 3.2±1.2 |
| CBS5206 | Grape must | South Africa | 32.2±5.6 |
| CBS5513 | Bantu-beer brewer | South Africa | 8.6±2.4 |
| UMY305 | Wine | Lombardy-Italy | 13.5±1.5 |
| UMY306 | Passito wine | Lombardy-Italy | 9.3±1.5 |
| UMY307 | Wine | Piedmont-Italy | 3.6±1.2 |
| UMY308 | Wine | Sicily-Italy | 4.0±2.2 |
| UMY309 | Wine | Lombardy-Italy | 15.2±1.4 |
| UMY310 | Wine | Lombardy-Italy | 3.6±0.4 |
| UMY311 | Grape must | Tuscany-Italy | 4.8±0.9 |
| UMY312 | Grape must | Tuscany-Italy | 3.3±0.9 |
| UMY313 | Grape must | Tuscany-Italy | 7.4±2.2 |
| UMY314 | Grape must | Tuscany-Italy | 10.8±2.2 |
| UMY315 | Grape must | Tuscany-Italy | 5.7±1.1 |
| UMY317 | Grape must | Tuscany-Italy | 31.6±2.1 |
| UMY318 | Grape must | Tuscany-Italy | 2.9±0.6 |
| UMY319 | Wine | Tuscany-Italy | 2.1±0.8 |
| UMY320 | Wine | Tuscany-Italy | 0.3±0.1 |
| UMY321 | Wine | Tuscany-Italy | 9.8±4.1 |
| UMY322 | Wine | Tuscany-Italy | 7.6±3.0 |
| UMY323 | Wine | Tuscany-Italy | 15.4±3.5 |
| UMY324 | Wine | Tuscany-Italy | 1.5±0.1 |
| UMY325 | Wine | Tuscany-Italy | 1.8±0.2 |
| UMY326 | Wine | Tuscany-Italy | 16.3±2.2 |
| UMY327 | Wine | Tuscany-Italy | 3.8±1.1 |
| UMY328 | Wine | Tuscany-Italy | 26.0±8.5 |
| UMY329 | Wine | Tuscany-Italy | 20.2±4.6 |
| UMY330 | Wine | Tuscany-Italy | 6.9±2.6 |
| UMY331 | Wine | Tuscany-Italy | 17.5±2.6 |
| UMY332 | Wine | Tuscany-Italy | 7.2±2.4 |
| UMY333 | Wine | Tuscany-Italy | 3.9±1.0 |
| UMY334 | Wine | Montenegro | 37.5±7.2 |
| UMY335 | Wine | Montenegro | 36.0±6.5 |
| UMY419 | Wine | Lombardy-Italy | 23.8±5.2 |
| UMY422 | Wine | Lombardy-Italy | 41.3±5.5 |
| UMY424 | Wine | Lombardy-Italy | 24.2±3.3 |
| UMY426 | Wine | Lombardy-Italy | 40.0±6.1 |
| UMY431 | Wine | Lombardy-Italy | 33.4±7.2 |
| UMY432 | Wine | Lombardy-Italy | 35.8±9.2 |
| UMY439 | Wine | Lombardy-Italy | 39.9±9.9 |
| 1 | Wine | Lombardy-Italy | 3.4±1.8 |
| 2 | Wine | Lombardy-Italy | 3.6±1.5 |
| 3 | Wine | Lombardy-Italy | 2.4±1.0 |
| 4 | Wine | Lombardy-Italy | 6.3±0.9 |
| 6 | Wine | Lombardy-Italy | 5.1±1.7 |
| 7 | Wine | Lombardy-Italy | 24.5±8.2 |
| 8 | Wine | Lombardy-Italy | 7.6±1.8 |
| 9 | Wine | Lombardy-Italy | 9.2±1.5 |
| 10 | Wine | Lombardy-Italy | 9.0±1.8 |
| 11 | Wine | Lombardy-Italy | 22.0±6.8 |
| 12 | Wine | Lombardy-Italy | 7.8±1.8 |
| 13 | Wine | Lombardy-Italy | 13.8±4.4 |
| 14 | Wine | Lombardy-Italy | 8.0±2.4 |
| 16 | Wine | Lombardy-Italy | 11.1±3.4 |
| 17 | Wine | Lombardy-Italy | 13.7±2.6 |
| 18 | Wine | Lombardy-Italy | 9.4±0.5 |
| 19 | Wine | Lombardy-Italy | 25.5±3.5 |
| 20 | Wine | Lombardy-Italy | 4.1±1.1 |
| 21 | Wine | Lombardy-Italy | 37.3±15.1 |
| 22 | Wine | Lombardy-Italy | 32.1±5.6 |
| 23 | Wine | Lombardy-Italy | 16.8±3.0 |
| 24 | Wine | Lombardy-Italy | 34.7±14.9 |
| 25 | Wine | Lombardy-Italy | 28.5±2.2 |
| 26 | Wine | Lombardy-Italy | 1.8±0.4 |
| 27 | Wine | Lombardy-Italy | 34.2±7.1 |
| 28 | Wine | Lombardy-Italy | 32.9±9.5 |
| 29 | Wine | Lombardy-Italy | 9.4±1.2 |
| 30 | Wine | Lombardy-Italy | 7.0±3.0 |
| 31 | Wine | Lombardy-Italy | 10.1±3.6 |
| 32 | Wine | Lombardy-Italy | 3.6±1.1 |
| 33 | Wine | Lombardy-Italy | 5.2±1.9 |
| 34 | Wine | Lombardy-Italy | 11.5±1.8 |
| 35 | Wine | Lombardy-Italy | 4.7±1.9 |
| 36 | Wine | Lombardy-Italy | 40.5±5.1 |
| 37 | Wine | Lombardy-Italy | 10.3±1.2 |
| 38 | Wine | Lombardy-Italy | 3.4±1.8 |
| 42 | Wine | Lombardy-Italy | 37.5±11.8 |
| 43 | Wine | Lombardy-Italy | 9.3±1.8 |
| 47 | Wine | Lombardy-Italy | 12.3±5.5 |
| 48 | Wine | Lombardy-Italy | 15.0±2.3 |
| 49 | Wine | Lombardy-Italy | 13.5±2.5 |
| 50 | Wine | Lombardy-Italy | 8.3±1.9 |
| 52 | Wine | Lombardy-Italy | 2.5±0.1 |

**Table S2.** *FLO1* and *FLO11* gene reconstructions in the two selected strains. The underlined sequences correspond to the overlapping regions and the bold underlined the restriction site of the *HaeIII* (GGCC) and *HhaI* (GCGC).

***FLO1*_CBS2499**

ATGATTTTTTCAAACGTTGTTAGAGTTTTGGCCATAACTTCCCTTTGCACAAAGGGTATGGCTGCTCCATATCCAGTAGCAAAAGGTGTAAAAGTTTACGAACGTGAGGCTGCACCAATTGCAGATCCAGAGCCGGTTGCTTTTACCTACAATGAAGAATTGCTAAAGAGAGACGGTCTTGCTAGTGCAATCAGTGCTCTTCTTACTGATGCTGTGAGCACAGTAGCCAATGGATTGCTTGGTACTCTTAACACATTGGTTCAAGGGCTAGTTAGTGATTTGACAAGCTGTGATGGTGTTCTTAATGATTTAGGTGCTGTTCTTGATGGTATCCTTACCGATGCTGGTGAAGGTGCTGACTCATCTGTTCAGGATGTTGTTACCCTTATTCAAAATCTTGTTGATGCCATCATTTCAGGTGCTTCCAGTGCAAATATTCTTACCGATTTATCTAATCTTCTAAGTGGAATTGTCTCAGCAGTTGGAACTGGTGGTGATGACGTTCTAACCACAGTGGGAAATCTTCTTGCAGAATTGGTTGAAAGTTTAACAACTGCCAAAGGTCAATGTTCTTCTACCGCCGCTGTTACCCTTACTGATGTTTGCGGAAATGCCATATCTTCTAGCTCTAGTACTTCTACTAATTTCGATGCAGAGGGCATTCTTGGAGATGTTGCCGAGATCATTAACGATGCAATCTCTTCTTTGACTTCAAGCACAGGCTCTGGAATACTTGGAGACGTTCTTGATTTGATTACAGAACTTATTGACGACTTGACTGACTGTGATGGTGTTTTGAATGATCTTGCTACTGATATCCAAAGCATTCTTTCAGAAGCAGGCTCCTCAGGAGATGATGTTCTTAGCGAAATTGCTAGTTTGCTTGGAGACCTCGTGACAGATATTCTTACCTCTGCCACTTCCCTTAATATATTAACTGACTTGAGTAACTTGGTCGGAAAAATCTTGAGTTCAGTTGGATCGTCAAGTAGTGACGTTCTTAGTGACATCGGTGATTTGCTTGAAAGTATTTTGAATACGCTTGCCAATGGTAGTGCATCTTGCTCATCAGAATCTTCTGCAAAGTCATCATCTTTATGTACCAGCTCAACAAACTCTCTTTCCAGCGCAAGCTCTAGCTCTGTGACTTCCTCCAGCTCTTCTGCCTCTACATCTTCTTCAAACTCCTCTGCTGAAACCACATCTGTCTCAAGCTTGACTGAATCTTCATCTGGTTCGAGCTCGACTGAGTCTACATCTGGTTCGAGCTCCGTTAGCGGTTCAACCGAAACATCATCTGCTACTGGTTCTAGCTCAGTTACTGAGTCAAGTGGATCAGCAAGTTCTACCGGCTCTAGCACTATAAGCGGATCGAGCACCGCATCGGGATCTGTCAATGGATCTGGTTCTAATGGATCAGGATCTAATTCGGAAAACGGTTCTAAGAATGGCTCGGGATCCGAATCAGGAAATGGATCGGGTTCAGGTTCAGGCTCAGGTGCTAACGGTAAGAATGTTGAAACAACTGTTATTACTGTTACATCATGCTCCGATCATGTCTGCTCTAAGACTCCTGTTACTACTGGCGTTTACTCTTACACTACAACTGAAAATGGTGTTGAAACCGTTTACACAACCTACTGCCCATTGACCTCTGAAGAGACTAAAGCAACTGTTTCTGCTTATCCAGAAGAGACTACTGTCATTACTGTCACCTCATGCTCTGATAATGTCTGCACTAAAATCCCTGTTACTACAGGTGTCTACTCTTACACAACAACCGAAAATGGTGCAGAGACCGTTTACACAACTTACTGCCCATTGACTGCCGAGGAGAATGAAGCAAGTGTTTCTGCTTCCCCAGAAACTTTAGCCTCAGTTTCATCATCCGCAGCTACTCAAATTGCGGCAGGCTCTGTTAGTGAGTACACAGGTGCATCTTCTTCGGCTCAATTGATTAAATCCTCAGCTTCATCTGTTGCTGCAGTCTCGACTTTGGCCTCATCAGCCGCTACAATTCAGCCTTCTGGCTCTGTTAGTGAGTACGAAGGAGCTGGCTCATCTATTCAATACGGAAGTCAGCTACTCTTAACATTGCTTGCTGTTTTCTTGTTATGA

***FLO11*_CBS2499**

ATGTTGGCATCCTTATTATTTGCTACTCTCGTTCAACTTGCAGAGGCATCCCCTATTGGCCTAAATATATTTGAAAAAAGGGAATTGGTCGCAGAATCCAAATGTCCTGATTGGAACTTTGATTGGAAATGTACACAGGGTAATTATCCATGTGCAACGTCCTCTGTTTCCAATGTCATTGCCTTAGGTAACAATGAATACCAAGTGACTTTCAACTTTGATGCTGATGGTTGTGCAGATCTTTCGAACTTAGGAGAATTGAAAGTGATTTCTCTTGATTCTCCAAATGGGAATGAGGATCTTTTGTTTTCAAGAAACTCAAACAAGATTACAAATATTGATCCATGTCACTGGTCGGCTACATTCGTACTTTATGGAGAAGATTATGGTGACTACATCTGTACACCATCATTTCAGGTTCAGTATGATTGGTTTTCTGGCAATGGCGTCTCAACTAGTGAAGAATCTTCCTGGAAATATACAGGTTCGTATGATTATCTCGTTGGTTGTAATGGTGATAATCAAGGACATTCAAATGCTGACTTCCCTCAGTACTGTTGGGTCACCTTCCTTGGGAATACATCAAGTTCTTCTAGTTCCTCCACAACCACCACAACTAGTTCTACTTCATCAACAACATCAACTTCATGCACTACATCAACTAGTTTAAGTACTACTTCGACGTCATCTACCCCTTCAACTACCACTCCATCTGCTTCAACTACTTCGAGCTCATCTACCTCATCAACTAGTACTTCATCTACTTCAACTTCCTCGAGCTCATCCACCTCATCAACTACTACTCCATCAACTTCAACTACTTTGACTTCATCTACCTCATCAACTTCATCCACTACATCAACTACTTCGAGCTCATCTACCTCATCAAGTACCACTCCATCCGCTTCGTCCACTTCATCGACTACTGGTTCAACTACCACTCCATCTTTTTCAACTACTTCGAGCTCATCAACTTCATCCACTACATCAACTAGTTTAAGTACTACTTCGACTTCATCTACCTCTTCAACTACCACTCCATCTACTTCAACTACTTCGAGCTCATCTACCTCATCAAGTACTATTCCATCTACTTTGTCCACTTCATCGACTACTAGTTCAACAACCACTCCATCTACCTCATCAACAAAGACTACTTTGAGTACTGCTTCTACTTCTAGTTCATCTTCTTCTTCGACTACTAGTTCGACCACTTCTCCATCCGCTTCGTCTACTTCATCGACTACTAGTTCAATTACCATTCCATCTACCTTATCAGCAAAAAATACTTCGAGTACAGACCCATCCACCCCGTCGACTACTAGTTTGAGTACTGCTTTATCCACTACATCTACAAAATCTAGTGTGAGTACCACTTCCACTTCATCCATCTCATCGACTACTATTTCGATTCCTACACCATCCACTTCCTTCACCGCTAGTTTGAGTAGTACTTCCACTTTGAGCTCAATTACAAGTTCAAGCACCAGTCCATCAATTTTATCTACGTCATCATCATTTAATTCCACCATCCCAACTACTAGTTGCGATACATTATCCAGCTCCTCAAAGTTGTCATCGGGCACCTCAGTTATTTCAGTTCCAACAACTTCTA**GCGC**ATCGTCTACAACAAATTCAGGTAACTTCTCATTAGGTTCCTCCACCATATTGAGCACTTCTAAGTTGCATTCATCGGCTTCTTCGAAATCATTGAGATTTTCCTCAAGTGAATCAACGATTTCTTCTGGTTATCTCACATCGACCATGCAAGCAACATTTTTGACTACGGTTACTGTTCCTTGCTCAACTTTGATCACGACAACAACATGTACCTTGAACAGATGTACTAGCTACGAGACGGCAGTTTCTACCAATAAAGTTGTTACCTTGGAAAGATCATCTGTTTTGCCTTCGAAATCTGCTAGTAAGTTATTATCATCCTCCATCACCGATGTTGTTCCAGCTACATCTTCGTCGGCATCGTTGAACTCAGTCTCAAAAGATCGCGAGACAACTTTCTTTACTACAGTGACTATTCCTTGCTCGACATTGATCACAACTACTACATGCACATGCACATTGAACAAATGTACCGCTTACCCAACAACTATTTCAACAAATTCTGTTGTCATTTCAGAAACGACTTCTAAGTTGCCTTTAGAAACTGCTTCTGAGCCAGCCACCTTGGTCACAAGCACAAAATTACCTGAAGAACCTAAATCCCAGACACCTTCTTCAGCTTCCACTACTGCAACTGTTTGCTCTGGAGAAACATGTTCAACTGCTCCAATTTCTCGTACTTCAAAGACTTCTGCAACAGAGTCAACGATAACGAGCAGTTCTTCCTCCGTGCACTCCACAGTTCAGTCAACTGCAAGCTCAACTTCGATTTCTGTGGCAATCAACAGTGCCATTCCATCTGCCTCTGTGTTTGTTGGAGGTGCCGGAACGTACACTCCAAACGTTTTTGTCGCATTTGTCATCAGTGCTATAGCATTTGTTATTTAA

***FLO1_*UMY321**

ATGATTTTTTCAAACGTTGTTAGAGTTTTGGCCATAACTTCCCTTTGCACAAAGGGTATGGCTGCTCCATATCCAGTAGCAAAAGGTGTAAAAGTTTACGAACGTGAGGCTGCACCAATTGCAGATCCAGAGCCGGTTGCTTTTACCTACAATGAAGAATTGCTAAAGAGAGACGGTCTTGCTAGTGCAATCAGTGCTCTTCTTACTGATGCTGTGAGCACAGTAGCCAATGGATTGCTTGGTACTCTTAACACATTGGTTCAAGGGCTAGTTAGTGATTTGACAAGCTGTGATGGTGTTCTTAATGATTTAGGTGCTGTTCTTGATGGTATCCTTACCGATGCTGGTGAAGGTGCTGACTCATCTGTTCAGGATGTTGTTACCCTTATTCAAAATCTTGTTGATGCCATCATTTCAGGTGCTTCCAGTGCAAATATTCTTACCGATTTATCTAATCTTCTAAGTGGAATTGTCTCAGCAGTTGGAACTGGTGGTGATGACGTTCTAACCACAGTGGGAAATCTTCTTGCAGAATTGGTTGAAAGTTTAACAACTGCCAAAGGTCAATGTTCTTCTACCGCCGCTGTTACCCTTACTGATGTTTGCGGAAATGCCATATCTTCTAGCTCTAGTACTTCTACTAATTTCGATGCAGAGGGCATTCTTGGAGATGTTGCCGAGATCATTAACGATGCAATCTCTTCTTTGACTTCAAGCACAGGCTCTGGAATACTTGGAGACGTTCTTGATTTGATTACAGAACTTATTGACGACTTGACTGACTGTGATGGTGTTTTGAATGATCTTGCTACTGATATCCAAAGCATTCTTTCAGAAGCAGGCTCCTCAGGAGATGATGTTCTTAGCGAAATTGCTAGTTTGCTTGGAGACCTCGTGACAGATATTCTTACCTCTGCCACTTCCCTTAATATATTAACTGACTTGAGTAACTTGGTCGGAAAAATCTTGAGTTCAGTTGGATCGTCAAGTAGTGACGTTCTTAGTGACATCGGTGATTTGCTTGAAAGTATTTTGAATACGCTTGCCAATGGTAGTGCATCTTGCTCATCAGAATCTTCTGCAAAGTCATCATCTTTATGTACCAGCTCAACAAACTCTCTTTCCAGCGCAAGCTCTAGCTCTGTGACTTCCTCCAGCTCTTCTGCCTCTACATCTTCTTCAAACTCCTCTGCTGAAACCACATCTGTCTCAAGCTTGACTGAATCTTCATCTGGTTCGAGCTCGACTGAGTCTACATCTGGTTCGAGCTCCGTTAGCGGTTCAACCGAAACATCATCTGCTACTGGTTCTAGCTCAGTTACTGAGTCAAGTGGATCAGCAAGTTCTACCGGCTCTAGCACTATAAGCGGATCGAGCACCGCATCGGGATCTGTCAGTGGATCAAGTATTACATCAGGTTCAGTTAGTGGATCAAGCACTGCCTCAAGTTCAGTTAGTGGATCAAGTATTACATCAGGTTCAGTTAGTGGATCAAGTACTGCATCAGGTTCAGTCAGTGGCTCTAACTCATTGACAGCTGCCACTGGTTCTTTAACCAAAACAAATGAAAGTGGTAACGAAACAAAGACATCATCAGGTGCAAATGCTAGTGCCACAGGTAATGGATCTGGTTCTAATGGATCTGGTTCTAATGGATCAGGATCTAATTCGGAAAACGGTTCTAAGAATGGCTCGGGATCCGAATCAGGAAATGGATCGGGTTCAGGTTCAGGCTCAGGTGCTAACGGTAAGAATGTTGAAACAACTGTTATTACTGTTACATCATGCTCCGATCATGTCTGCTCTAAGACTCCTGTTACTACTGGCGTTTACTCTTACACTACAACTGAAAATGGTGTTGAAACCGTTTACACAACCTACTGCCCATTGACCTCTGAAGAGACTAAAGCAACTGTTTCTGCTTATCCAGAAGAGACTACTGTCATTACTGTCACCTCATGCTCTGATAATGTCTGCACTAAAATCCCTGTTACTACAGGTGTCTACTCTTACACAACAACCGAAAATGGTGCAGAGACCGTTTACACAACTTACTGCCCATTGACTGCCGAGGAGAATGAAGCAAGTGTTTCTGCTTCCCCAGAAACTTTAGCCTCAGTTTCATCATCCGCAGCTACTCAAATTGCGGCAGGCTCTGTTAGTGAGTACACAGGTGCATCTTCTTCGGCTCAATTGATTAAATCCTCAGCTTCATCTGTTGCTGCAGTCTCGACTTTGGCCTCATCAGCCGCTACAATTCAGCCTTCTGGCTCTGTTAGTGAGTACGAAGGAGCTGGCTCATCTATTCAATACGGAAGTCAGCTACTCTTAACATTGCTTGCTGTTTTCTTGTTATGA

***FLO11*_UMY321**

ATGTTGGCATCCTTACTATTTGCTACTCTCGTTCAACTTGCAGAGGCATCCCCTATTGGCCTAAATATATTTGAAAAAAGGGAATTGGTCGCAGAATCCAAACCAATGTCCAATGATTGGAACTTTGATTGGAAATGTACACAGGGTAATTATCCATGTGCAACGTCCTCTGTTTCCAATGTCATTGCCTTAGGTAACAATGAATACCAAGTGACTTTCAACTTTGATGCTGATGGTTGTGCAGATCTTTCCAACTTAGGAGAATTGAAAGTGATTTCTCTTGATTCTCCAAATGGGAATGAGGATCTTTTGTTTTCAAGAAACTCAAACAAGATTACAAATATTGATCCATGTCACTGGTCGGCTACATTCGTACTTTATGGAGAAGATTATGGTGACTACATCTGTACACCATCATTTCAGGTTCAGTATGATTGGTTTTCTGGCAATGGCGTCTCAACTAGTGAAGAATCTTCCTGGAAATATACAGGTTCGTATGATTATCTCGTTGGTTGTAATGGTGATAATCAAGGACATTCAAATGCTGACTTCCCTCAGTACTGTTGGTCACCTTCCTCGGATACATCAAGTTCTTCTAGTTCCTCTACAACCACCACAACTAGTTCTACTTCATCAACAACATCAACTTCATCCACTACATCAACTAGTTTAAGGACTACTTCGACTTCATCTACCTCTTCAACTACCACTCCATCTACTTCAACTACTTCGAGCTCATCTACCTCATCAAGTGCTATTCCATCCACTTTGTCCACTTCATCGACTACTAGTTCGACAACCACTCCATCTACCTCATCAACAAAGACTACTTTGAGTACTGCTTCTACTTCTAGTTCATCTTCTTCTTCGACTACCAGTTCAACAACCACTCCATCAACTTCAACTACTTCGAGCTCATCTACCTCATCAAGTACTATTCCATCTACTTTGTCCACTTCGTCGACTACTAGTTCGACAACCACTCCATCTACCTCATCAACAAAGACTACTTTGAGTACTGCTTCTACTTCTAGTTCATCTTCTTCTTCGACTACCAGTTCAACAACCACTCCATCAACTTCAACTACTTCGAGCTCATCTACCTCATCAAGTACTATTCCATCTACTTTGTCCACTTCGTCGACTACTAGTTCAACAACCACTCCATCTACCTCATCAACAAAGACTACTTTGAGTACTGCTTCTACTTCTAGTTCATCTTCTTCTTCGACTACCAGTTCAACAACCACTCCATCAACTTCAACTACTTCGAGCTCATCTACCTCATCAAGTACTATTCCATCTACTTTGTCCACTTCGTCGACTACTAGTTCAACAACCACTCCATCTACCTCATCAACAAAGACTACTTTGAGTACTGCTTCTACTTCTAGTTCATCTTCTTCTTCGACTACTAGTTCAACAACCACTCCATCAACTTCAACTACTTCGAGCTCATCTACCTCATCAAGTACTATTCCATCTACTTTGTCCACTTCATCGACTACTAGTTCAACAACCACTCCATCTACCTCATCAACAAAGACTACTTTGAGTACTGCTTCTACTTCTAGTTCTTCTTCTTCTTCGACTACTAGTTCGACCACTTCTCCATCCACTTCGTCTACTTCATCGACTACTAGTTCAATTACCATTCCATCTACCTTATCAGCAAAAAATACTTCGAGTACAGACCCATCCACCCCGTCGACTACTAGTTTGAGTACTGCTTTATCCACTATATCTACAAAATCTAGTGTGAGTACCACTTCCACTTCATCTATTCCATCTACCATATCAGCAAAAAATACCTCGAGTACAGACCCATCCACCCCGTCGACTACTAGTTTGAGTACTGCTTTATCCACTACATCTACAAAATCTAGTGTGAGTACCACTTCCACTTCATCCATCTCATCGACTACTATTTCGATTCCTACACCATCCACTTCCTTCACCGCTAGTTTGAGTAGTACTTCCACTTTGAGCTCAATTACAAGTTCAAGCACCAGTCCATCAATTTTATCTACGTCATCATCATTTAATTCCACCATCCCAACTACTAGTTGCGATACATTATCCAGCTCCTCAAAGTTGTCATCGGGCACCTCAGTTATTTCAGTTCCAACAACTTCTAGCGCATCGTCTACAACAAATTCAGGTAACTTCTCATTAGGTTCCTCCACCATATTGAGCACTTCTAAGTTGCATTCATCGGCTTCTTCGAAGTCATTGAGATTTTCCTCAAGTGAATCAACGATTTCTTCTGGTTATCTCACATCGACCATGCAAGCAACATTTTTGACTACGGTTACTGTTCCTTGCTCAACTTTGATCACGACAACAACATGTACCTTGAACAGATGTACTAGCTACGAGACGGCAGTTTCTACCAATAAAGTTGTTACCTTGGAAAGATCATCTGTTTTGCCTTCGAAATCTGCTAGTAAGTTATTATCATCCTCCATCACCGATGTTGTTCCAGCTACATCTTCGTCGGCATCGTTGAACTCAGTCTCAAAAGATCGCGAGACAACTTTCTTTACTACAGTGACTATTCCTTGCTCGACATTGATCACAACTACTAAATGCACATTGAACAAATGTACCGCTTACCCAACAACTATTTCAACAAATTCTGTTGTCATTTCAGAAACGACTTCTAAGTTGCCTTTAGAAACTGCTTCTGAGCCAGCCACCTTGGTCACAAGCACAAAATTACCTGAAGAACCTAAATCCCAGACACCTTCTTCAGCTTCCACTACTGCAACTGTTTGCTCTGGAGAAACATGTTCAACTGCTCCAATTTCTCGTACTTCAAAGACTTCTGCAACAGAGTCAACGATAACGAGCAGTTCTTCCTCCGTGCACTCCACAGTTCAGTCAACTGCAAGCTCAACTTCGATTTCTGTGGCAATCAACAGTGCCATTCCATCTGCCTCTGTGTTTGTTGGAGGTGCCGGAACGTACACTCCCAACGTTTTTGTCGCATTTGTCATCAGTGCTATAGCATTTGTTATTTAA

**Table S3.** Percentage of identity of the variable regions of *FLO1* and *FLO11* genes among the 14 selected strains*.*

|  | AWRI1499 | CBS1943 | CBS73 | UCD2041 | CBS2499 | UMY321 | AWRI1613 | CBS2796 | CBS74 | CBS4459 | UMY334 | UMY320 | CBS5206 | UMY309 |
| --- | --- | --- | --- | --- | --- | --- | --- | --- | --- | --- | --- | --- | --- | --- |
|  | ***FLO1*** | | | | | | | | | | | | | |
| AWRI1499 | 100.0 | - | - | - | - | - | - | - | - | - | - | - | - | - |
| CBS1943 | 77.3 | 100.0 | - | - | - | - | - | - | - | - | - | - | - | - |
| CBS73 | 77.1 | 100.0 | 100.0 | - | - | - | - | - | - | - | - | - | - | - |
| UCD2041 | 77.8 | 98.9 | 98.9 | 100.0 | - | - | - | - | - | - | - | - | - | - |
| CBS2499 | 78.1 | 99.2 | 99.2 | 99.2 | 100.0 | - | - | - | - | - | - | - | - | - |
| UMY321 | 78.1 | 99.2 | 99.2 | 99.2 | 100.0 | 100.0 | - | - | - | - | - | - | - | - |
| AWRI1613 | 78.1 | 99.2 | 99.2 | 99.2 | 100.0 | 100.0 | 100.0 | - | - | - | - | - | - | - |
| CBS2796 | 78.1 | 99.2 | 99.2 | 99.2 | 100.0 | 100.0 | 100.0 | 100.0 | - | - | - | - | - | - |
| CBS74 | 78.1 | 99.2 | 99.2 | 99.2 | 100.0 | 100.0 | 100.0 | 100.0 | 100.0 | - | - | - | - | - |
| CBS4459 | 77.9 | 99.2 | 99.2 | 99.2 | 100.0 | 100.0 | 100.0 | 100.0 | 100.0 | 100.0 | - | - | - | - |
| UMY334 | 77.8 | 99.2 | 99.2 | 99.2 | 100.0 | 100.0 | 100.0 | 100.0 | 100.0 | 100.0 | 100.0 | - | - | - |
| UMY320 | 77.8 | 99.2 | 99.2 | 99.2 | 100.0 | 100.0 | 100.0 | 100.0 | 100.0 | 100.0 | 100.0 | 100.0 | - | - |
| CBS5206 | 78.2 | 94.3 | 94.3 | 95.4 | 94.9 | 94.9 | 94.9 | 94.9 | 94.9 | 94.9 | 94.8 | 94.8 | 100.0 | - |
| UMY309 | 79.6 | 93.7 | 93.7 | 94.8 | 94.5 | 94.5 | 94.5 | 94.5 | 94.5 | 94.5 | 94.5 | 94.5 | 97.5 | 100.0 |
|  | ***FLO11*** | | | | | | | | | | | | | |
| AWRI1499 | 100.0 | - | - | - | - | - | - | - | - | - | - | - | - | - |
| CBS1943 | 85.2 | 100.0 | - | - | - | - | - | - | - | - | - | - | - | - |
| CBS73 | 85.5 | 99.1 | 100.0 | - | - | - | - | - | - | - | - | - | - | - |
| UCD2041 | 81.8 | 89.9 | 89.9 | 100.0 | - | - | - | - | - | - | - | - | - | - |
| CBS2499 | 86.5 | 99.4 | 99.5 | 91.1 | 100.0 | - | - | - | - | - | - | - | - | - |
| UMY321 | 78.2 | 80.2 | 80.5 | 76.7 | 79.6 | 100.0 | - | - | - | - | - | - | - | - |
| AWRI1613 | 78.2 | 80.7 | 81.1 | 80.3 | 82.7 | 96.8 | 100.0 | - | - | - | - | - | - | - |
| CBS2796 | 85.0 | 99.4 | 99.5 | 88.9 | 98.2 | 79.2 | 80.0 | 100.0 | - | - | - | - | - | - |
| CBS74 | 85.0 | 99.2 | 99.8 | 89.1 | 98.3 | 79.3 | 80.2 | 98.0 | 100.0 | - | - | - | - | - |
| CBS4459 | 70.6 | 98.3 | 98.0 | 91.3 | 97.3 | 69.5 | 72.6 | 95.9 | 96.7 | 100.0 | - | - | - | - |
| UMY334 | 83.2 | 85.0 | 85.0 | 80.6 | 84.4 | 93.9 | 95.8 | 83.8 | 84.9 | 72.0 | 100.0 | - | - | - |
| UMY320 | 75.8 | 79.1 | 79.0 | 74.9 | 77.6 | 98.7 | 95.5 | 78.1 | 78.2 | 68.4 | 94.3 | 100.0 | - | - |
| CBS5206 | 77.8 | 82.7 | 83.2 | 78.7 | 83.1 | 95.7 | 95.7 | 82.9 | 82.5 | 74.0 | 92.1 | 94.6 | 100.0 | - |
| UMY309 | 84.6 | 99.4 | 99.5 | 89.3 | 98.6 | 79.6 | 80.4 | 99.6 | 98.9 | 97.8 | 84.8 | 78.7 | 82.5 | 100.0 |

**Table S4.** CBS2499 strain results of the Box-Behnken experiment: flocculation percentages (F%) and fold change (in terms of 2^-ΔΔCt^ values) of *FLO1* and *FLO11* genes in the different growth conditions*.*

| RUN | pH | Ethanol (%) | mSO_2_ (mg/L) | F% | *FLO1* (fold change) | *FLO11* (fold change) |
| --- | --- | --- | --- | --- | --- | --- |
| 1 | 3.5 | 8.75 | 0 | 52.4±6.5​ | 0.62 | 0.43 |
| 2 | 3.5 | 8.75 | 0.25 | 53.5±6.4​ | 0.78 | 0.51 |
| 3 | 4.5 | 8.75 | 0 | 73.1±5.9​ | 0.24 | 0.37 |
| 4 | 4.5 | 8.75 | 0.25 | 35.4±7.2​ | 0.37 | 0.60 |
| 5 | 4 | 5 | 0 | 69.8±4.4​ | 0.38 | 0.76 |
| 6 | 4 | 5 | 0.25 | 28.9±7.8​ | 0.46 | 0.96 |
| 7 | 4 | 12.5 | 0 | 35.7±7.3​ | 0.65 | 1.27 |
| 8 | 4 | 12.5 | 0.25 | 80.2±4.2​ | 1.17 | 1.26 |
| 9 | 3.5 | 5 | 0.125 | 63.6±5.7​ | 0.04 | 0.35 |
| 10 | 4.5 | 5 | 0.125 | 49.3±5.2​ | 0.19 | 0.56 |
| 11 | 3.5 | 12.5 | 0.125 | 84.9±4.1​ | 1.21 | 4.18 |
| 12 | 4.5 | 12.5 | 0.125 | 30.7±8.6​ | 0.35 | 2.74 |
| 13 | 4 | 8.75 | 0.125 | 72.6±5.5​ | 0.18 | 0.75 |
| 14 | 4 | 8.75 | 0.125 | 71.1±5.8​ | 0.21 | 0.49 |
| 15 | 4 | 8.75 | 0.125 | 74.3±5.4​ | 0.10 | 0.50 |
| Permissive condition | 4.5 | 5 | 0 | 70.9±6.1​ | Calibrator | Calibrator |
